# Fungal diversity and seasonal succession in ash leaves infected by the invasive ascomycete *Hymenoscyphus fraxineus*

**DOI:** 10.1111/nph.14204

**Published:** 2016-09-26

**Authors:** Hugh Cross, Jørn Henrik Sønstebø, Nina E. Nagy, Volkmar Timmermann, Halvor Solheim, Isabella Børja, Håvard Kauserud, Tor Carlsen, Barbara Rzepka, Katarzyna Wasak, Adam Vivian‐Smith, Ari M. Hietala

**Affiliations:** ^1^ Norwegian Institute of Bioeconomy ResearchPb. 115ÅsNO‐1431Norway; ^2^ Department of BiosciencesSection for Genetics and Evolutionary BiologyUniversity of OsloPb. 1066 BlindernOsloNO‐0316Norway; ^3^ Faculty of Chemistry UJJagiellonian UniversityIngardena 3Kraków30‐060Poland; ^4^ Department of Pedology and Soil GeographyInstitute of Geography and Spatial ManagementJagiellonian UniversityGronostajowa 7Kraków30‐387Poland

**Keywords:** ash dieback, *Hymenoscyphus fraxineus*, indigenous fungi, internal transcribed spacer (ITS), invasive pathogens, metabarcoding

## Abstract

- High biodiversity is regarded as a barrier against biological invasions. We hypothesized that the invasion success of the pathogenic ascomycete *Hymenoscyphus fraxineus* threatening common ash in Europe relates to differences in dispersal and colonization success between the invader and the diverse native competitors.
- Ash leaf mycobiome was monitored by high‐throughput sequencing of the fungal internal transcribed spacer region (ITS) and quantitative PCR profiling of *H. fraxineus* DNA.
- Initiation of ascospore production by *H. fraxineus* after overwintering was followed by pathogen accumulation in asymptomatic leaves. The induction of necrotic leaf lesions coincided with escalation of *H. fraxineus* DNA levels and changes in proportion of biotrophs, followed by an increase of ubiquitous endophytes with pathogenic potential.
- *H. fraxineus* uses high propagule pressure to establish in leaves as quiescent thalli that switch to pathogenic mode once these thalli reach a certain threshold – the massive feedback from the saprophytic phase enables this fungus to challenge host defenses and the resident competitors in mid‐season when their density in host tissues is still low. Despite the general correspondence between the ITS‐1 and ITS‐2 datasets, marker biases were observed, which suggests that multiple barcodes provide better overall representation of mycobiomes.

## Introduction

A continental scale dieback threatens the future existence of common ash (*Fraxinus excelsior*) and poses a set of cascading impacts upon the biodiversity associated with this keystone tree species in Europe (Pautasso *et al*., 2013; Mitchell *et al*., 2014). Dieback of common ash is caused by the invasive ascomycete *Hymenoscyphus fraxineus* (syn. *H. pseudoalbidus*, anamorph *Chalara fraxinea*) (Kowalski, 2006; Queloz *et al*., 2011; Baral *et al*., 2014). In Asia, the presumed native range, this fungus has been regarded as a leaf saprophyte of Manchurian ash (*F. mandshurica*) (Zhao *et al*., 2013; Han *et al*., 2014; Zheng & Zhuang, 2014), which is a close relative of common ash (Wallander, 2008). Recent studies indicate that the fungus is a leaf endophyte of Manchurian ash (Cleary *et al*., 2016) and can also show some pathogenic potential in its native range (Drenkhan *et al*., 2016).

Dieback of common ash was first recorded in Poland in the early 1990s (Przybył, 2002). Since 2001, an intensive spread of the disease has been observed in central, northern, eastern and western Europe (Juodvalkis & Vasiliauskas, 2002; Przybył, 2002; Kowalski & Łukomska, 2005; Lygis *et al*., 2005; Kowalski, 2006; Timmermann *et al*., 2011), and only populations at the southern and eastern range margins of common ash currently remain disease‐free (McKinney *et al*., 2014).

The pathogen has the ability to colonize the compound leaf, shoots, main stem and even the roots of common ash (Kirisits & Cech, 2009; Kowalski & Holdenrieder, 2009; Schumacher *et al*., 2010). Young trees often die within a few years of infection, while older trees become chronically diseased and susceptible to secondary diseases such as root rot caused by the white‐rot fungi *Armillaria* (Skovsgaard *et al*., 2010). Ash trees are affected by the disease not only in the forest, but also in nurseries, on roadsides, in associated plantations, and in parks and gardens.

*Hymenoscyphus fraxineus* is an outcrossing heterothallic fungus, and the airborne ascospores have a significant role in primary infection and long‐distance dispersal (Bengtsson *et al*., 2012; Gross *et al*., 2012a,b, 2014b *;* Kraj *et al*., 2012; Hamelin *et al*., 2016). During the epidemic stage, *H. fraxineus* shows the ability to simultaneously liberate ascospores on massive scales early in the morning (Timmermann *et al*., 2011; Hietala *et al*., 2013), indicating that propagule pressure may be an important strategy for colonization success. According to the current model, *H. fraxineus* ascospores germinate on the leaf surface, giving rise to mycelia that spread to the leaf petiole and further into connecting stem tissues to cause shoot dieback (Gross *et al*., 2014a). The invasive behavior of *H. fraxineus* is obviously intimately linked with efficient pathogen capture of the leaf vein system, its primary sporulation substrate and main niche also in its native range in Asia. By contrast, ash shoot infection by *H. fraxineus* can be considered a dead‐end in the life cycle of this fungus, because its ascomata are rarely formed on twigs and stems of common ash (Gross *et al*., 2014b). The nutritional modes of *H. fraxineus* in common ash leaves remain to be clarified. Several studies have suggested that local biodiversity represents an important line of defense against the spread of invaders (e.g. Kennedy *et al*., 2002; Bairey *et al*., 2016).

Fungal community studies have been strengthened in recent years through a combination of high‐throughput sequencing (HTS) and a well‐curated database of fungal internal transcribed spacer (ITS) sequences (Kõljalg *et al*., 2013). DNA metabarcoding studies have shown that highly diverse fungal communities are associated with healthy leaves of other angiosperm trees (Jumpponen & Jones, 2009; Cordier *et al*., 2012; Bálint *et al*., 2013; Voříšková & Baldrian, 2013) , and we predicted that already during early summer, before the sporulation period of *H. fraxineus*, leaves of common ash would be exposed to high colonization pressure by a wide range of functionally divergent fungi. There is increasing evidence that propagule pressure is an important ecological trait that influences the success of introduction as well as the transition of invasive species to subsequent stages of local establishment, spreading outside the area of introduction and eventual widespread dominance (Lockwood *et al*., 2005; Colautti *et al*., 2006). We hypothesized that the ability of *H. fraxineus* to capture the leaf vein system is a result of advantages in propagule pressure and colonization strategies. To test these hypotheses, compound ash leaf samples were collected throughout the growing season in two consecutive years from a stand exhibiting an epidemic level of ash dieback. Leaflet and petiole tissues were subjected to quantitative PCR (qPCR) profiling of *H. fraxineus* DNA content and to HTS of fungal sequences separately amplified from the ITS‐1 and ‐2 of the rDNA gene cluster. As reference, airborne fungal spores captured from the experimental stand during the peak sporulation period of *H. fraxineus* were also subjected to HTS. Our data reinforce the fact that, during the ascospore production period, *H. fraxineus* accumulates in ash leaves as quiescent thalli that switch to the pathogenic growth phase once the initial tissue colonization reaches a specific threshold. The massive mid‐season sporulation provides a crucial signal from the saprophytic phase and enables this fungus to challenge host defense and the resident competitors when their density in host tissues is still low.

## Materials and Methods

### Plant and spore material

Randomly chosen compound leaves from the understory of common ash trees were sampled throughout the growing season in 2011 and 2012 in a stand located 30 km south of Oslo (Ås municipality, 59°40′44″N, 10°46′31″E,100 m above sea level (asl)), exhibiting epidemic levels of ash dieback.

In 2011, leaves were sampled from three selected trees on a weekly basis from the beginning of July until the leaves shed at the end of August; altogether 27 compound leaves were collected. In that year the monthly precipitation in June and July in the experimental region was 20–60 mm above the long‐term averages for the area, while the mean temperatures were similar to (June) or slightly above (July) the long‐term averages for the area (Supporting Information Fig. S1).

**Figure 1:**
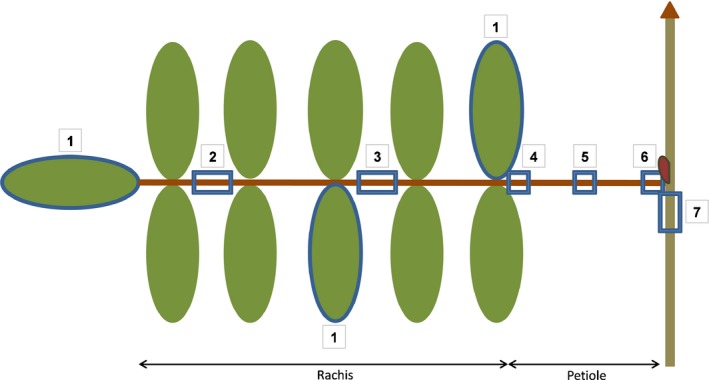
Tissue samples (shown as blue frames) taken from the compound ash leaf. 1, leaflets (three leaflets, apical, central and basal, pooled together); 2, rachis, upper; 3, rachis, middle; 4, petiole, upper; 5, petiole, middle; 6, petiole, base; 7, twig.

In 2012, 12 trees were sampled from the end of June until the period of leaf shed in mid‐September. These consisted of six healthy appearing trees and six that had shoot symptoms of ash dieback in the beginning of the season (Fig. S2). As an addition, the twig region directly below the axillary bud of the sampled compound leaves was also collected (Fig. 1). Altogether 50 compound leaf/twig samples were collected. In 2012 the monthly precipitation for June and July in the experimental region was 10–30 mm above the long‐term average for the area, whereas the mean temperatures were 1–2.5°C below the average (Fig. S1).

DNA from airborne spores, collected by a volumetric Burkard spore sampler at the experimental stand at four time‐points in June and August 2010 in our previous study (Hietala *et al*., 2013), were used as reference material in sequencing.

### DNA isolation and qPCR

For each tree and sampling time, the apical leaflet, one leaflet from the middle, and one from the base of the compound leaf were excised and pooled together to form a balanced sample. In addition, separate, *c*. 5‐mm‐long samples were taken from the upper and middle rachis and likewise from the upper, middle and basal parts of the petiole. In 2012 the subsampling was modified, omitting the middle part of petiole, and taking a sample of the twig region directly below the axillary bud of the sampled compound leaves (Fig. 1). Samples were processed separately and the FW of each sample was recorded for normalization of qPCR data. Dissected and subsampled ash tissues were stored at −20°C until DNA extraction.

Leaflet samples were frozen with liquid nitrogen and pulverized with a mortar and pestle, while the other tissues were pulverized in liquid N_2_‐chilled Eppendorf tubes with a Retsch 300 mill (Retsch Gmbh, Haan, Germany). Up to 50 mg tissue was processed with DNeasy Plant Mini Kit (Qiagen) according to the manufacturer's instructions, to a final elution volume of 50 μl.

The real‐time PCR quantification of *Hymenoscyphus fraxineus* (T. Kowalski) Baral, Queloz, Hosoya was performed as described by Ioos *et al*. (2009) (see Methods S1). The primer and probe concentrations per assay were 300 and 100 nM, respectively (Ioos *et al*., 2009). A standard curve with known concentrations of *H. fraxineus* DNA was prepared from pure cultures as previously described (Hietala *et al*., 2013). To ensure that the cycle threshold values from the experimental samples were within the standard curves, and that PCR inhibitory compounds potentially present in undiluted samples remained low in the assay, a 3‐log dilution series was prepared so that for each sample the undiluted DNA and the 10‐ and 100‐fold dilutions were used as templates for real‐time PCR. Standard curves were constructed by plotting the *Ct* values against log‐transformed DNA amounts. The calculated linear regression equation was used for interpolation of pathogen DNA amount in unknown samples.

Many of the undiluted leaf and twig tissue DNA samples showed a higher *Ct* value than the 10‐fold diluted template, whereas the differences in *Ct* values between 10‐ and 100‐fold dilutions were in the range obtained for the log dilutions of the corresponding standard curve samples. Thus, many undiluted templates appeared to be compromised by PCR inhibitory compounds. Therefore, *Ct* values from the 10‐fold diluted templates were used in subsequent calculations. Sampling time‐specific differences in *H. fraxineus* DNA amount were tested by ANOVA and Fisher's least significant difference (LSD) *post hoc* test, and considered statistically significant at *P *<* *0.05.

### Pyrosequencing of ITS1 region

DNA extracted from the leaflet, petiole upper and petiole base tissues collected in 2011 and the reference spore material was subjected to 454 pyrosequencing following the protocol of Lindner *et al*. (2013). DNA from the leaf tissue samples collected from three selected trees was pooled at equimolar concentrations so that one DNA sample per tissue type and sampling time was processed: altogether, 27 pooled DNA samples (one leaflet, one petiole upper and one petiole base sample for each of the nine sampling dates) were analyzed. This entailed a nested PCR approach, using the fungal‐specific primers ITS1F and ITS4 (White *et al*., 1990; Gardes & Bruns, 1993) in the first step and fusion primers ITS5 and ITS2 (White *et al*., 1990) in the nested step, using 50 ×  diluted product from the first PCR. Fusion primers contained 16 different 10 bp unique tags and 454 pyrosequencing adaptors A and B to both ITS5 and ITS2, respectively (see Methods S1 for details). PCR products were normalized to a single DNA concentration using the SequelPrep Normalization Plate (Invitrogen) and then cleaned with Wizard_SV PCR Clean‐Up System (Promega). GS FLX sequencing of the tagged amplicons was performed at the Norwegian High‐Throughput Sequencing Centre (http://www.sequencing.uio.no) using one 454 plate divided into eight compartments. We included two negative controls through all analyses from the DNA extraction step.

### Ion Torrent PGM sequencing of ITS2 region

The same 27 pooled DNA samples from leaf tissues processed for ITS‐1 sequencing were also used for ITS‐2 sequencing, along with the reference spore material. The fungal ITS‐2 region was amplified using the degenerate gITS7 primer (Ihrmark *et al*., 2012) together with the ITS4 primer (White *et al*., 1990; Methods S1). Each reaction was cleaned with 1.1 volumes of Ampure XP (Beckman Coulter Inc., Pasadena, CA, USA), and the products were ligated to barcoded adaptors as outlined in the Ion Amplicon Library Preparation user guide and Ion Xpress fragment kit (PN 4468326 rev B and P/N 4471252, respectively), and where the A adaptor contained a sample specific Ion Xpress_barcode identification sequence (Thermo Fisher Scientific, Waltham, MA, USA; catalogue no. 4471250). The resulting products were pooled in equal amounts and purified using another round of Ampure XP cleaning, and analyzed on a Bioanalyzer 2100 High Sensitivity DNA Chip (Agilent Biosciences, Santa Clara, CA, USA). Subsequently library pools were diluted and sequenced according to Ion Torrent manufacturer specifications (Thermo Fisher Scientific) on the Ion PGM using 314 v2 chips with 400 bp chemistry. Sequences were inspected using FastQC (Andrews, 2010).

### Extrapolation of total fungal biomass in ash leaf tissues

The total DNA amount of all fungi present in ash leaf tissues was extrapolated using the following formula: total fungal DNA amount (ng) = (Hfrax_DNA_ × 100%)/Hfrax_seq,_ where Hfrax_DNA_ is the *H. fraxineus* DNA amount (ng) determined by qPCR, and Hfrax_seq_ is the corresponding ITS‐2 sequence proportion (%) of *H. fraxineus* in the sample at a given time.

### Bioinformatics and statistical analyses

#### Processing of raw sequences

All sequence reads from 454 and Ion Torrent were processed using the programs Cutadapt (Martin, 2011) and FastX‐Toolkit (http://hannonlab.cshl.edu/fastx_toolkit/index.html) in a pipeline with custom bash and python scripts (Methods S1). Briefly, all adapter and primer sequences were trimmed at both ends of each sequence, and then filtered for minimum length (100 bp) and quality (at least 90% of reads with a phred score of 20). All files were renamed and converted to the FASTA format for downstream analyses, and lodged with the NCBI Sequence Read Archive (ID PRJNA305543).

#### Operational taxonomic unit (OTU) clustering and taxonomy assignment

The overall method for clustering reads and assigning taxonomy followed a modified and customized version of open‐reference OTU clustering, as described by Rideout *et al*. (2014), as this method has been found to be a good compromise between OTU stability and the inclusion of novel taxa (He *et al*., 2015). The analyses proceeded in three major steps. Initially, all sequence reads were dereplicated and then clustered into OTUs using the program Swarm (Mahé *et al*., 2015); these OTUs were used as queries for searching against the UNITE‐INSD fungal ITS and NCBI nt databases, and the hits from these searches were used to construct a reference sequence database. In the second phase, all reads were clustered with these reference sequences to assign taxonomy (‘closed reference clustering’) using Usearch v.8 (Edgar, 2010) at minimum 97% similarity. In the last step, any reads that did not cluster closely with the reference sequences (33.5% of ITS‐1 and 39.5% of ITS‐2 reads) were reclustered into OTUs using Usearch (‘open reference clustering’); taxonomy for these OTUs was assigned using the Usearch utax algorithm and RDP method in Qiime (Caporaso *et al*., 2010). Taxonomy for open reference clustering was limited to the genus level to avoid inflation of rare species/OTUs as a result of sequence error or gaps in the sequence database. For details of this approach, see Methods S1.

#### Statistical and taxonomic analyses

The program Qiime (Caporaso *et al*., 2010) was used to summarize taxonomic tables and plot relative abundance of taxa across all samples, and by date and tissue type. Abundance tables were imported into the program Megan, v.5.10.3 (Huson *et al*., 2011), to map and chart the taxonomy, and compare the ITS‐1 and ITS‐2 results. Alpha rarefaction curves were calculated in Megan by repeatedly subsampling the dataset (1000 replicates) and computing the number of taxa in the subsample. All OTU sequence read totals for which taxonomy could be assigned at least to genus level were combined by genus for ordination analyses. The sample totals for the 50 most abundant genera were normalized using the CSS method (Paulson *et al*., 2013) in Qiime. In order to determine gradients of taxonomic composition, we conducted principal component analyses (PCA) on normalized OTU abundance tables using the R package vegan (Oksanen *et al*., 2016). To consider sampling time and tissue type‐specific differences in numbers of genera, as well as differences in genus read proportions, and positions along PC1 and PC2, we applied one‐way ANOVA with the LSD *post hoc* test, with *P *≤* *0.05 using the SPSS 22.0 (IBM Inc., Armonk, NY, USA). Pearson product moment correlation coefficients were calculated between the qPCR and the ITS‐2 datasets for selected taxa.

All *Hymenoscyphus* reads were remapped to a single representative sequence (FJ597975, type specimen of *H. fraxineus*) using the BWA mem algorithm (Li & Durbin, 2009). Additionally, reads assigned to other taxa of relatively high abundance (> 1 or 2%) were extracted and compared with reference sequences and OTUs to determine their relative species composition and diversity. These reads and references were aligned using Geneious v.6 (Kearse *et al*., 2012) for comparison.

## Results

### Accumulation of pathogen DNA in ash tissues

**Figure 2:**
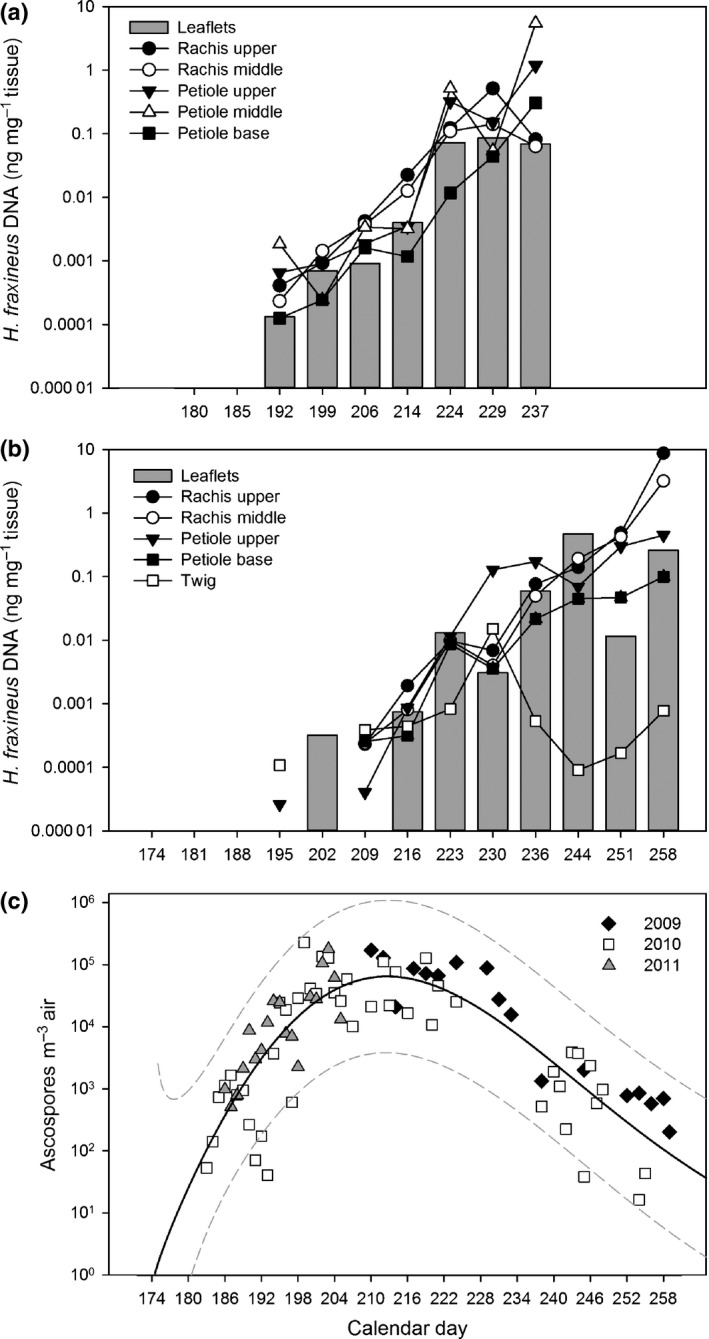
*Hymenoscyphus fraxineus* DNA amount in ash tissues (ng DNA mg^–1^ tissue) sampled throughout the summers of 2011 (a) and 2012 (b), and the amount of airborne pathogen ascospores at the experimental stand during 2009–2011 (c), either analyzed by a real‐time PCR assay specific to the DNA of the fungus or by microscopy (spore data). For leaflet tissues from 2011 and spores, the data are obtained from Hietala *et al*. (2013). Calendar days with missing values in (a) and (b) indicate that the fungus was not detected. Note that, for spores, the sampling covered only part of the sporulation season in 2009 and 2011. In panel (c) the continuous line indicates a model fitted to the data, while the dashed lines show 95% predictive intervals calculated as described in Supporting Information Methods S1.

In 2011 necrotic lesions on leaf veins were induced during the first week of August. The presence of *H. fraxineus* DNA in all ash leaf tissues was first detected by qPCR on 11 July (day 192), after which the pathogen DNA level showed a generally continuous increase (Fig. 2a), which coincided with the vigorous increase of airborne ascospores of *H. fraxineus* at the stand in July (Fig. 2c). The first significantly higher pathogen DNA levels compared with those observed on 11 July (day 192) occurred on leaflets on 18 July (day 199), on 2 August (day 214) for the upper part of the rachis and on 12 August (day 224) for the remaining leaf tissues. The largest fold‐changes between two consecutive sampling times occurred between 2 and 12 August (days 212–224) when the pathogen DNA showed between five‐ and 161‐fold increases (up to 18‐fold increase in leaflet, petiole base, and rachis tissues, and 90–161‐fold increase in the upper and middle petioles); excluding the upper part of the rachis and petiole base, these increments were statistically significant.

In 2012, the amount of necrotic leaf lesions increased rapidly after the second week of August in all trees. *H. fraxineus* DNA was first detected in all sampled tissues on the 3 August (day 216; Fig. 2b); the first significantly higher pathogen DNA levels compared with those observed on 3 August occurred on leaflets on 10 August (day 223) and, for the remaining leaf tissues, during the period between 23 August and 7 September (days 236 and 251). Twig tissues showed a unique pattern in pathogen DNA accumulation in which the peak on 17 August (day 230), differing significantly from that on 3 August, was followed by a steep decline. There was no clear relationship between the pathogen DNA titer in leaf tissues and the general health of the tree over the course of the season (Figs S2, S3).

### Fungal community profiling by ITS‐1

After processing of raw reads and filtering for quality and length, there were a total of 98 113 ITS‐1 sequences from 454 sequencing (average of 2803 per sample over 31 samples). About 66.5% of the total reads clustered with 342 reference sequences, and the remaining proportion of reads were *de novo*‐clustered into 969 OTUs.

**Figure 3:**
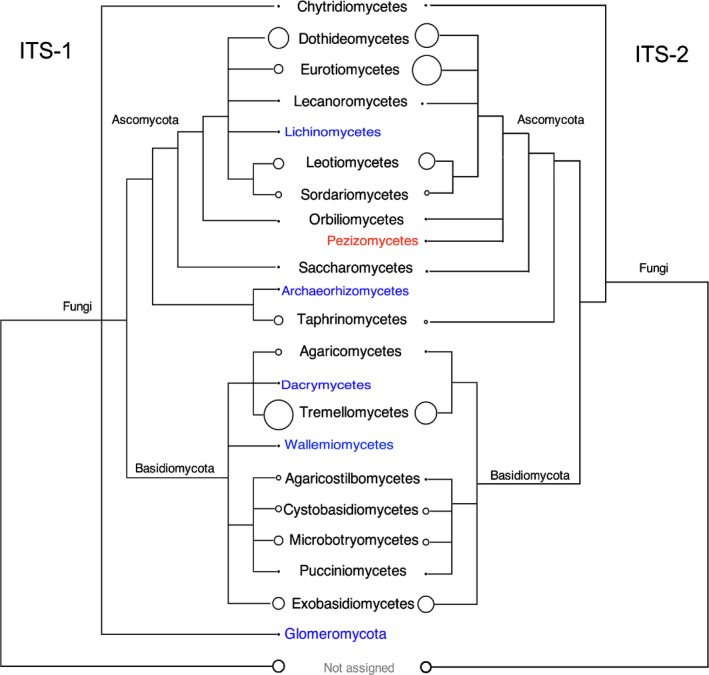
Overview of fungal taxonomic diversity of internal transcribed spacer‐1 (ITS‐1) and ITS‐2 results, at the class taxonomic rank as visualized with the Megan software, and lined up by taxon. The size of the circle at the tips indicates the relative number of reads assigned to each sample. Classes in blue indicate those groups that were found in ITS‐1 but not in ITS‐2, and taxa in red indicate those found in ITS‐2 but not in ITS‐1.

The vast majority of sequences was associated with fungi, with a small proportion (5.7%) left unassigned. A very small fraction of reads (< 0.3%) were identified as plants (mostly the host species *Fraxinus excelsior*), algae, and protists. All subsequent analyses were confined to fungal taxa. Although there were smaller numbers of reads in some individual leaf samples (including leaflet, and petiole upper and lower parts), rarefaction analyses across most samples suggest that the number of reads sequenced had sampled most of the species diversity (Fig. S4a). The taxonomic assignments of ITS‐1 sequences revealed a fungal community comprising a wide range of basidiomycete and ascomycete taxa (Figs 3, S5).

**Figure 4:**
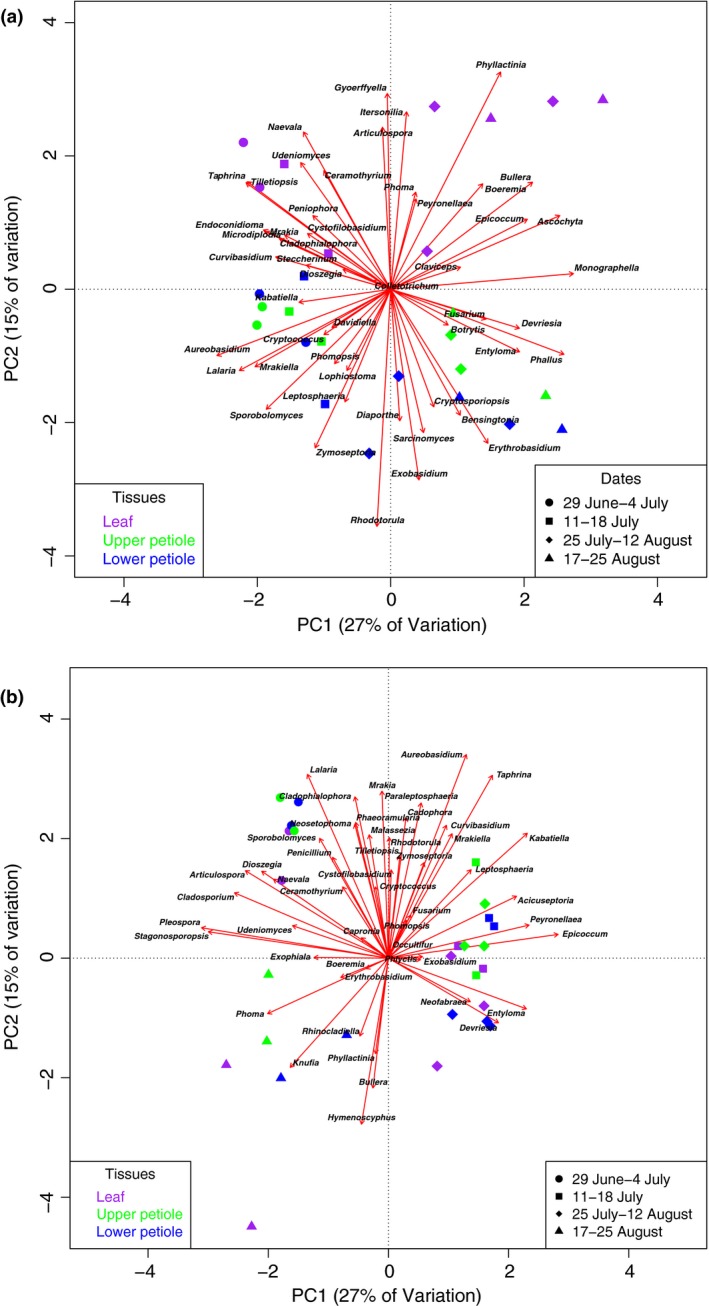
Principal component analysis (PCA) biplots of 50 most abundant genera of internal transcribed spacer‐1 (ITS‐1) (a) and ITS‐2 (b) datasets. The samples are indicated by symbols and colors, with the symbols corresponding to the time periods (circles, sampling dates 29 June and 4 July; squares, 11 and 18 July; diamonds, 25 July and 2 and 12 August; triangles, 17 and 25 August). Colors indicate the tissue type of each sample (purple for leaf, green for upper petiole, and blue for lower petiole). The placement of generic names indicates the samples with which they are correlated (e.g. *Hymenoscyphus* is correlated with late season samples in ITS‐2 (b)). The arrows pointing to each genus represent eigenvectors showing the correlation of one taxon to another; genera with a small angle between their vectors are strongly positively correlated, genera with angles at 180° are expected to be strongly negatively correlated, and genera perpendicular to each other (angles of 90 or 270°) are not correlated to each other.

Overall, most differences were observed between spore and plant tissues (Tables S1–S3). The PCA analysis of ITS‐1 data from leaf tissues indicated significant differences in fungal species composition, both between all time categories (*P *<* *0.0001), and between leaflet and petiole tissues (*P *<* *0.005) (Fig. 4a). The arrows on the PCA biplot (Fig. 4a) indicate a range of species highly correlated with each other (Oksanen *et al*., 2016).

Despite the high taxonomic diversity across all samples, a striking omission was that almost no reads were assigned to *Hymenoscyphus* species, in stark contrast with both the qPCR data and the visual observation of disease symptoms. Examination of reference sequences showed a very large insert in the ITS‐1 region of this genus. Therefore we suspected that the PCR product size resulting from the ITS5/ITS2 primer pair (581 bp) for *H. fraxineus* was too long for efficient sequencing and that this was the primary cause of low *Hymenoscyphus* read numbers.

### Fungal community profiling by ITS‐2

As *Hymenoscyphus* sequences were rarely detected with the ITS5/ITS2 primer pair, ITS‐2 amplicons were sequenced using the gITS7/ITS4 primers with the Ion Torrent PGM, as the product size is 280 bp for *H. fraxineus*, well within the range for Ion Torrent and about the average size for fungal species. Ion Torrent sequencing produced 177 681 ITS‐2 reads after filtering (average of 5923 per sample over 30 samples). The overall results were similar to ITS‐1: 60.5% of reads clustered with 481 reference sequences, and the remaining sequences were clustered into 1539 *de novo* OTUs. The majority of taxonomic matches were to fungi, with a small fraction matching plants or algae (< 1%), and 3.7% were left unassigned.

The measures of alpha and beta diversity were similar to ITS‐1, exhibiting a highly diverse fungal community with a wide range of ascomycetes and basidiomycetes in each sample, and sufficient read coverage (Figs 3, 4, S4b). Between samples, the primary differences were found between leaf tissues and spores (Tables S1, S2, S4). The PCA analysis of ITS‐2 data from leaf tissues indicated significant differences in fungal species composition between all time categories (*P *<* *0.006), but not between leaflet and petiole tissues (Fig. 4b). The PCA biplot shows correlation between taxa (Fig. 4b). Taxa that were abundant late in the season, such as the genera *Phyllactinia* and *Phoma*, were positively correlated with *Hymenoscyphus*, while taxa that were more abundant early in the season, such as *Taphrina*,* Tilletiopsis*,* Cladophialophora*, were negatively correlated with *Hymenoscyphus*.

All the *c*. 13 000 reads identified as *Hymenoscyphus* were aligned and evaluated in the Geneious software program. While the most common genotype showed 100% sequence similarity to the *H. fraxineus* type specimen in ITS‐2 (Fig. S6), two base pair changes in one variant were in sufficient quantity to identify a distinct *H. fraxineus* assignment. Across most samples, the putative additional genotype is near the frequency of that of the reference genotype (Fig. S7).

### Seasonal and tissue‐specific patterns in sequence abundance of indigenous fungal species

**Figure 5:**
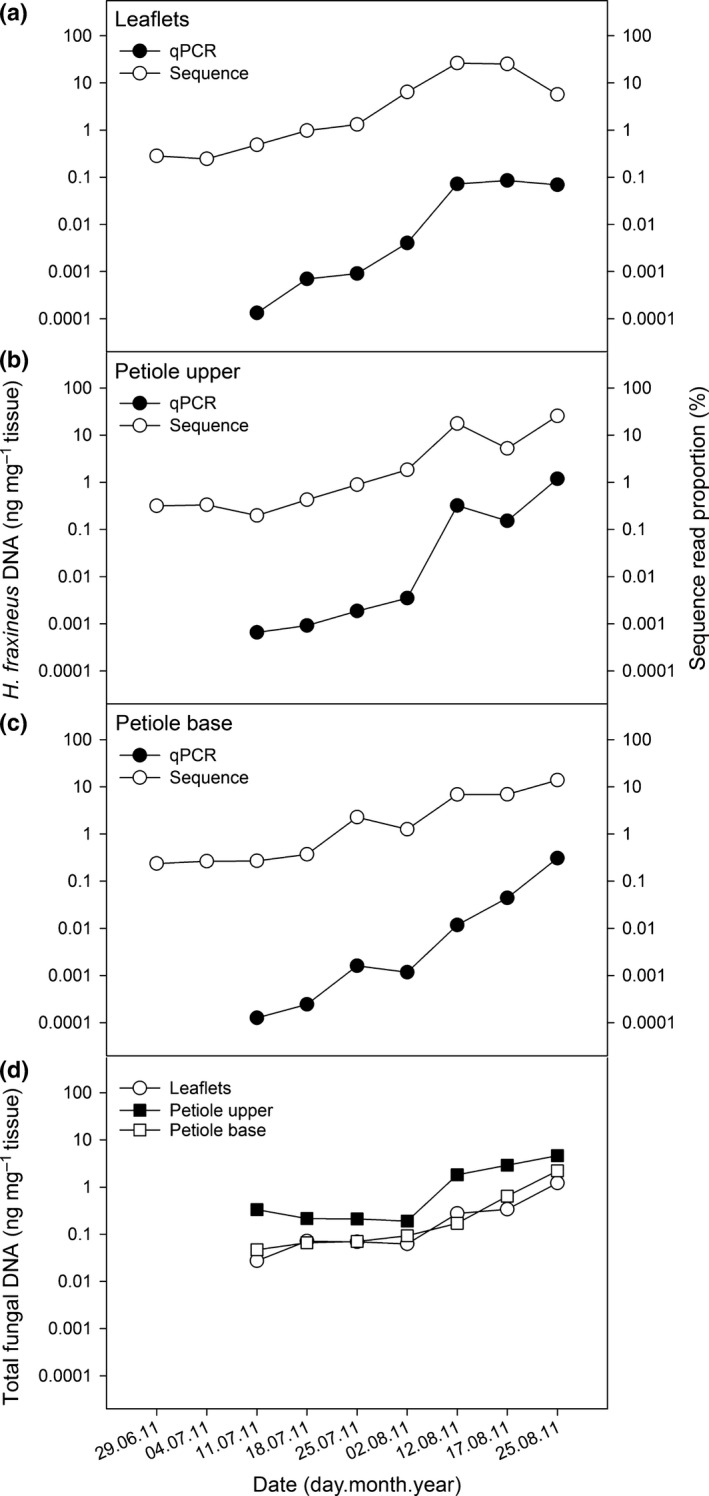
Comparison of *Hymenoscyphus fraxineus* DNA amount estimates as determined by real‐time PCR (qPCR) and sequencing of internal transcribed spacer‐2 (ITS‐2) region (sequence) for ash leaflet (a), petiole upper (b) and petiole base (c) samples collected in 2011, and estimates of total fungal DNA amount in the three leaf tissue types (d).

There was a strong correlation between the *H. fraxineus* ITS‐2 sequence percentages and qPCR‐based DNA amounts (Fig. 5a–c): the Pearson product moment correlation coefficients between sample datasets for the leaflet, the upper part of the petiole and the petiole base were 0.83, 0.93 and 0.88, respectively. Extrapolation of total fungal DNA amount in the leaf material from 2011 (Fig. 5d) suggested that no major change took place in July, whereas the total fungal biomass increased rapidly in all leaf tissues after 2 August, and that by the end of August the petiole tissues hosted more fungal biomass than the leaflets.

There was a good overall correspondence between the ITS‐1 and ‐2 datasets as both generally showed a similar seasonal pattern for a given genus (Table S1) – the disparities between the ITS‐1 and ITS‐2 datasets were primarily related to the relative abundance of basidiomycetes in the order Tremellales, (Fig. S5a), and ascomycetes in the orders Taphrinales, (Fig. S5b), Chaetothyriales and Helotiales (Fig. S5c).

**Figure 6:**
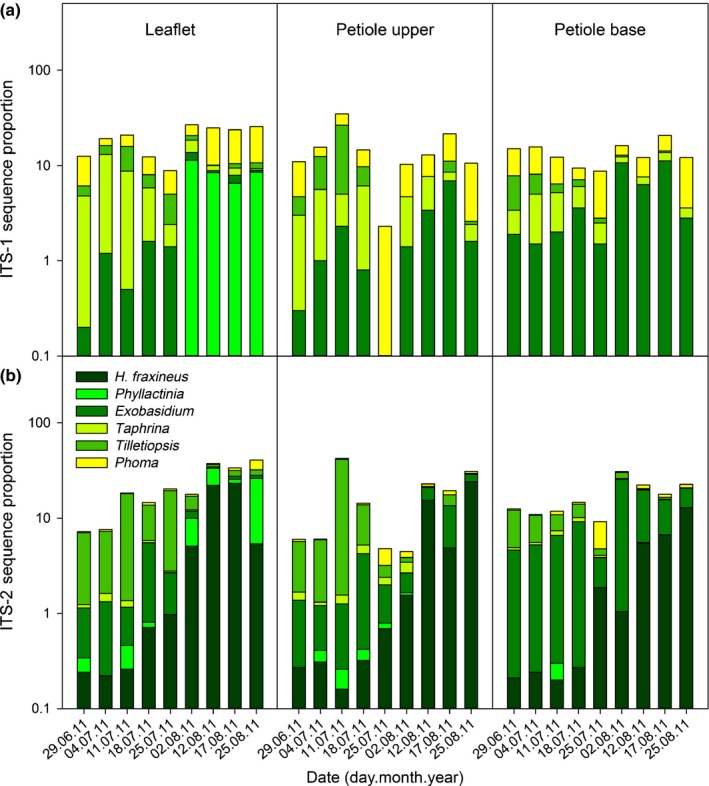
Seasonal changes in internal transcribed spacer‐1 (ITS‐1) (a) and ITS‐2 (b) read proportions of *Hymenoscyphus fraxineus*, and dominant biotrophic (*Phyllactinia*,* Exobasidium*,* Taphrina*), epiphytic (*Tilletiopsis*) and endophytic (*Phoma* anamorph/related teleomorph genera) fungi in ash leaf tissues.

The vast majority of fungi associated with ash leaves showed essentially stable read percentages throughout July (Fig. 6; Tables S1, S2). As exceptions, towards the end of July *H. fraxineus* showed significant increases in ITS‐2 read percentages in leaflets and petiole base, the biotrophic genus *Exobasidium* showed significant increase in ITS‐1 and ITS‐2 read proportions in leaflets, while the biotrophic genus *Phyllactinia* showed a nonsignificant trend of increase in ITS‐1 and ‐2 read percentages in leaflets. By contrast, the biotrophic genus *Taphrina* showed a significant decline in ITS‐1 read percentages in leaflets towards the end of July, while the epiphyte genus *Tilletiopsis* showed a general decline in ITS‐1 and ITS‐2 read percentages in petiole tissues.

Most of the significant changes in ITS read percentages of fungi associated with ash leaves occurred in August. Besides *H. fraxineus*, both epiphytic yeasts (*Bullera*,* Rhodotorula*), biotrophs (*Exobasidium*,* Phyllactinia*) and endophytes with pathogenic potential (*Boeremia*,* Diaporthe*,* Epicoccum*,* Fusarium*,* Knufia*,* Phoma*,* Pleospora*) showed significant increases in ITS‐1 or ‐2 read percentages in one or several leaf tissues (Fig. 6; Tables S1, S2). Genera that showed a significant decline in ITS‐1 or ‐2 read proportions towards the end of summer in one or several leaf tissues included *Aureobasidium*,* Tilletiopsis*,* Sporobolomyces* and *Taphrina* (Table S1).

Fungi that, across the season, showed significant positive correlation with ITS‐2 sequence proportions of *H. fraxineus* in one or several leaf tissues included *Exobasidium*,* Phyllactinia*,* Devriesia*,* Knufia* and *Phoma*, whereas the genera *Aureobasidium*,* Tilletiopsis, Sporobolomyces* and *Taphrina* showed significant negative correlations with ITS‐2 sequence proportions of *H. fraxineus* (Table S5).

Regarding tissue specificity, the majority of fungi showed relatively similar read percentages between the leaflet and upper and basal parts of the petiole at any given time (Tables S1, S2). However, fungi in the ascomycetous genera *Naevala*,* Gyoerffyella* and *Phyllactinia* showed, across the season, generally higher read percentages in leaflet than in the petiole tissues. Fungi that showed generally higher read percentages in one or both of the petiole tissues than in the leaflet included basidiomycetes in the genera *Exobasidium*,* Rhodotorula* and *Phallus* and ascomycetes in the genus *Leptosphaeria*.

Less than 40% of the fungal genera detected by ITS‐1 or ITS‐2 sequencing in leaf tissues in the period 11 July–12 August 2011 were also detected in the sequenced spore samples that had been captured at the experimental stand by a volumetric air sampler in the previous year (Tables S3, S4). *Tilletiopsis* was by far the most common genus in both datasets and showed a decline in sequence proportion in the spore material towards the end of the sampling period, similar to that observed in leaf material. Regarding ITS‐2, the stably high read proportions of *H. fraxineus* in the spore material at the end of July and beginning of August coincided with a drastic increase in its read proportions in leaf tissues. The maximum ITS‐2 read proportions of genera *Cladosporium* and *Cladophialophora* in the spore material at the end of the sampling period were in contrast to the decline observed in their read proportions in leaf tissues. Biotrophs (*Exobasidium*,* Phyllactinia*) generally showed a trend of increase in read proportions in the spore material towards the end of the sampling period, which was in line with their increased read proportions in leaf tissues towards autumn. At class level, the average ITS‐1 and ITS‐2 sequence proportions for sporulating endophytic genera in Dothideomycetes (*Cladosporium* excluded), Eurotiomycetes (*Cladophialophora* excluded), Helotiales (*H. fraxineus* excluded) and Sordariomycetes were below 0.5%.

## Discussion

Dieback of common ash is caused by the invasive ascomycete *Hymenoscyphus fraxineus*, which is considered to originate from Asia. The disease was first observed in Europe in the 1990s and is currently threatening the existence of common ash across Europe. An increase in population density is a prerequisite for introduced species to become widespread and dominant in a new environment, and the current profiling of fungal community structure in ash leaves increases our understanding of life cycle traits that contribute to the invasiveness of *H. fraxineus* in Europe. Through a combination of approaches, we tracked the colonization pressure of the pathogen and indigenous fungi in ash leaf tissues, documenting the establishment of first contact between the tree, indigenous fungi and the pathogen, the following quiescent phase, and finally the intraspecific fungal competition upon colonization of weakened host tissues.

### Methodological considerations

The utility of metabarcoding for accurately estimating species abundances is unclear, as studies have found both a strong correlation (Amend *et al*., 2010a) and little correlation (Deagle & Tollit, 2007; Pompanon *et al*., 2012) between number of reads and species abundance. In our study, the absence of *H. fraxineus* sequences from the ITS‐1 data as a result of its large insertion serves as an extreme example of barcode length bias affecting the results, and controlling for this variable produced accurate predictions for *Hymenoscyphus* at least. Both ITS‐1 and ITS‐2 are associated with many potential biases (Aird *et al*., 2011; Ihrmark *et al*., 2012; Blaalid *et al*., 2013; Lindahl *et al*., 2013; Tedersoo *et al*., 2015; Wang *et al*., 2015), and these seem to have a much greater effect than differences in HTS platform (Yergeau *et al*., 2012). In our study, the correlation between ITS‐1 and ITS‐2 was generally high for abundant species, although it varied for specific fungal groups. Our results demonstrate that multiple barcode markers provide a more complete representation of the fungal community.

### Changes in fungal community structure during the asymptomatic phase

Sporulation of *H. fraxineus* usually starts at the end of June at the ash stand under study (Hietala *et al*., 2013). Our data indicated a continuous accumulation of pathogen biomass in ash leaves from the initiation of sporulation throughout the season. As symptoms of necrotic leaf lesions were first observed at the beginning of August, the interaction period of *H. fraxineus* with leaves of common ash clearly involves a long latent phase.

Already in the symptomless period, leaves of common ash hosted a phylogenetically and functionally highly diverse mycobiota: epiphytic fungi comprised mostly yeasts, obligatory parasites that depend on living plant tissue to complete their life cycle, and endophytes comprising many facultatively parasitic filamentous ascomycetes (Fig. S5). The same functional groups were documented in HTS studies of leaf‐associated fungal communities in bur oak (*Quercus macrocarpa*; Jumpponen & Jones, 2009), European beech (*Fagus sylvatica*; Cordier *et al*., 2012), sessile oak (*Quercus petraea;* Voříšková & Baldrian, 2013) and balsam poplar (*Populus balsamifera*; Bálint *et al*., 2013).

The strong correlation between the increments of ITS‐2 sequence read proportions of *H. fraxineus* and the qPCR‐based pathogen DNA amount estimates across the season implies that changes in the biomass of *H. fraxineus* in ash leaf tissues were more pronounced than those concerning coinhabiting fungi. Among co‐associated fungi, only the biotrophic genus *Exobasidium* showed a significant increase in read proportion during the asymptomatic phase, whereas the biotrophic genus *Taphrina* showed a significant decline. The peaking of the genus *Taphrina* early in the growing season was also observed in leaves of bur oak (Jumpponen & Jones, 2010).

The general invariability of read percentages of fungi in July would suggest that during the asymptomatic phase *H. fraxineus* accumulates in leaf tissues as quiescent epiphytic and endophytic thalli that do not induce major physiological changes in host tissue or interact with other fungi to an extent that would influence the general stability of the fungal community.

### Changes in fungal community during the symptomatic phase

In 2011 the amount of airborne *H. fraxineus* ascospores at the experimental stand reached a maximum by mid‐July. Necrotic lesions in ash leaves occurred during the first week of August, coincident with a strong increase in *H. fraxineus* DNA level and ITS‐2 read proportions in leaflets. The high concentrations of *H. fraxineus* DNA detected on necrotic leaf tissues in comparison to green ash leaf tissues suggest a cause–effect relationship (Steinböck, 2013). The genus *Hymenoscyphus* belongs to the order Helotiales, which includes many species that shift between endophytic and pathogenic growth phases, and Sieber (2007) postulated that once the density of endophytes exceeds a certain tissue‐specific threshold, the endophytic thalli resume growth and kill the host tissues.

The total fungal biomass in leaf tissues was extrapolated from *H. fraxineus* qPCR quantities and ITS‐2 sequence proportions, because *H. fraxineus* has a generally average genome size for fungi (http://www.zbi.ee/fungal-genomesize). However, the copy number of ITS rDNA gene cluster can vary between species, and therefore these results need to be considered as relative and rough estimates. Similar variation in species‐specific conversion factors also applies to the more conventional fungal biomass assays based on chitin and ergosterol assays (e.g. Eikenes *et al*., 2005). The extrapolated vigorous increase in total fungal biomass in ash leaf tissues after formation of necrotic leaf lesions during the first week of August in 2011 implies that a range of fungi resumed growth in weakened tissues. The fungi that showed significant increases in read percentages in one or several leaf tissues after the first week of August included saprophytic epiphytes (*Bullera*) and biotrophs (*Phyllactinia*) (Fig 4b), coincident also with their read percentage increases in the spore samples towards autumn. The powdery mildew fungus *Phyllactinia fraxini* is a widespread associate of common ash and other ash species (Braun, 1995). The other fungi that increased significantly in read percentages in one or several leaf tissues after the first week of August included endophytes having pathogenic potential (*Fusarium*,* Pleospora*, the anamorph genus *Phoma* and related teleomorph genera *Boeremia* and *Epicoccum*), but these were hardly detected in the spore material. The amount of fungal biomass typically increases in leaves of deciduous trees towards autumn (e.g. Voříšková & Baldrian, 2013). Before ash dieback appeared in Germany, fungal isolations throughout the season from healthy leaves of common ash showed a trend of increase in isolation frequency of these endophytes towards autumn (Reiher, 2011). In the current epidemic stage of ash dieback, the foliage of all ash trees at a stand become obviously infected by *H. fraxineus*, and it is difficult to assess to what extent a late‐summer increase in biomass of an endophyte is triggered by natural host senescence. The increase of read proportions of resident endophytes directly after the escalation of *H. fraxineus* biomass in leaves and the formation of leaf necrotic lesions is presumably at least partly triggered by host tissue weakening by *H. fraxineus*.

Dimorphic ascomycetes in the genus *Aureobasidium* are common epiphytes in the phyllosphere of trees, common ash included (Sláviková *et al*., 2007), and tend to increase in frequency across the season (e.g. Jumpponen & Jones, 2010). In the present study, this genus showed a significant decline in sequence read percentages in the leaflet and petiole base after the first week of August. The most detailed studies on the association of *Aureobasidium species* with trees are in relation to apple, where these fungi show increased colonization on leaf veins across the season (McGrath & Andrews, 2006). While their spatial localization on ash leaves remains to be established, a primary localization on leaf veins could mean competition with the vein specialist *H. fraxineus*, and could account for the observed decline in sequence proportion of this genus.

### The significance of primary inoculum in the invasiveness of *H. fraxineus*

The observed high propagule pressure of *H. fraxineus* is typical of invasive species (Simberloff, 2009; Hamelin *et al*., 2016). The low presence of functionally related native endophytes in the spore material during the peak sporulation period of *H. fraxineus*, and the general increase of sequence proportions of these fungi in leaf tissues right after the development of necrotic lesions are consistent with a life cycle that involves early‐season establishment by a small primary inoculum followed by a quiescence phase, eventual resumption of growth, and production of secondary inoculum in weakened host tissues. Species of *Aureobasidium*,* Phoma* and *Fusarium* have been detected in buds of common ash during winter (Chen, 2011), suggesting that their primary inoculum to leaf infection may originate from propagules that overwinter in meristematic tissues. Based on our DNA level profiling of *H. fraxineus in planta*, the capacity of this fungus to produce symptoms on leaves depends on a large inoculum. In this respect, the aggressiveness of this fungus may well be comparable to many common ash endophytes, but it is presumably compensated by the huge primary inoculum of this invader. This would be in line with the propagule pressure theory (Lockwood *et al*., 2005) that is used to explain the success of invasive species. Sustained investment in the mid‐season production of ascopores may enable *H. fraxineus* to overcome defense responses of common ash and to challenge the resident endophyte competitors, whose density in leaf tissues is still low at this time in summer. We propose that seasonal and quantitative differences in sporulation between *H. fraxineus* and indigenous competitors facilitate the invasiveness of this pathogen. There are very few other studies available that have compared the propagule pressure of invasive fungal plant parasites and their native competitors. When monitoring the spread of *Heterobasidion irregulare*, a North American conifer pathogen introduced to central Italy, Garbelotto *et al*. (2010) concluded that a seasonal difference in spore production between this invader and a native competitor facilitates the establishment and spread of this alien species. While mate limitation impacts the spreading rate of outcrossing fungal plant pathogens (Hamelin *et al*., 2016), the equal frequency of occurrence of the two mating types in European populations of *H. fraxineus* (Gross *et al*., 2012b) has obviously facilitated the rapid spread of *H. fraxineus*.

### The role of diseased and asymptomatic ash individuals in the pathogen life cycle

To examine the relationship between tree health condition and pathogen growth, in 2012 we sampled leaves from ash trees with no crown symptoms and trees that showed shoot dieback. Both phenotypes showed comparable rates of pathogen DNA accumulation in leaf tissues, suggesting that the difference in the degree of shoot symptoms cannot be a result of tree‐specific variation in pathogen leaf colonization. As *H. fraxineus* ascomata form on overwintered leaf tissues, this implies that symptom‐free trees also support the build‐up of infection pressure within a forest stand, an observation that may have ramifications for ash management strategies in Europe. Considering the apparently low aggressiveness of *H. fraxineus*, it seems likely that, following a local introduction, several years are required to build up a propagule pressure that is sufficient for this fungus to cause disease. Such a scenario would explain the wave‐like spread of ash dieback at the invasion frontier (e.g. Hamelin *et al*., 2016).

### Future prospects

According to the current model, infection of ash leaf tissues by *H*. *fraxineus* ascospores is followed by mycelial spread through the petiole into twigs and shoots to cause shoot dieback (Gross *et al*., 2014a, and references therein). In the unusually cold summer of 2012, accumulation of *H. fraxineus* DNA in leaf tissues appeared delayed by 2–3 wk compared with 2011, presumably because of delayed onset of sporulation. It remains to be clarified how variation between years and different climatic regions affect propagule pressure and success of shoot infection by *H. fraxineus*.

The huge propagule pressure exerted by *H. fraxineus* can be envisaged to result in leaf colonization by a large number of genets. This scenario is supported by the fairly similar frequency of two ITS‐2 sequence variants of *H. fraxineus* in all ash leaf tissues by the end of the season. The territorial behavior of *H. fraxineus* is manifested as vegetative incompatibility in nonself confrontations (Brasier & Webber, 2013). Besides interactions with indigenous fungi, the interactions between the different genets of *H. fraxineus* need to be explored in order to increase further our understanding of factors that contribute to invasion success of this pathogen.

## Author contributions

V.T., N.E.N., I.B., H.S. and A.M.H. designed the research and collected the material, T.C. and H.K. performed ITS‐1 sequencing and J.H.S. and A.V‐S. performed ITS‐2 sequencing. B.R. and K.W. performed DNA extraction and qPCR. H.C. carried out all bioinformatics analyses, and, together with N.E.N. and A.M.H., performed the statistical analyses. H.C. and A.M.H. wrote the first version of the manuscript and revised it based on comments from all other co‐authors.

## Supporting information

Please note: Wiley Blackwell are not responsible for the content or functionality of any Supporting Information supplied by the authors. Any queries (other than missing material) should be directed to the *New Phytologist* Central Office.

**Fig. S1** Monthly precipitation and mean temperature in 2011 and 2012.
**Fig. S2** Damage assessment of sampled ash trees in early (22 June) and late (23 August) summer 2012.
**Fig. S3** *Hymenoscyphus fraxineus* DNA amount in tissues collected from healthy and diseased ash trees throughout the summer 2012 and analyzed by a qPCR assay specific to the DNA of the fungus.
**Fig. S4** Alpha rarefaction curves of individual samples for next generation sequencing ITS‐1 and ITS‐2 datasets.
**Fig. S5** Seasonal changes in ITS‐1 and ITS‐2 read proportions of fungal taxa at the experimental stand.
**Fig. S6** Portion of nucleotide alignment of *Hymenoscyphus* ITS‐2 sequences including a putative new undescribed genotype of *H*. *fraxineus*.
**Fig. S7** Seasonal changes in *H. fraxineus* DNA amount, determined by qPCR and by ITS‐2 read percentages of the two main sequence variants assigned to this species.
**Table S1** The most common fungal genera present in both ITS‐1 and ‐2 datasets, and their sequence proportions (%) in leaf tissues in early summer (29 June–11 July), mid‐summer and late summer (12–25 August) in 2011
**Table S2** The most common fungal genera detected in one ITS dataset only and their sequence proportions in leaf tissues in early summer (29 June–11 July), mid‐summer (18 July–02 August) and late summer (12–25 August) in 2011
**Table S3** ITS‐1 sequence percentages of fungi detected in air samples captured by a volumetric spore sampler at the experimental ash stand in 2010
**Table S4** ITS‐2 sequence percentages of fungi detected in air samples captured by a volumetric spore sampler at the experimental ash stand in 2010
**Table S5** Pearson product moment correlation between ITS‐2 sequence proportions of *Hymenoscyphus fraxineus* and the most common fungal general in different ash leaf tissues across the season 2011
**Methods S1** Detailed laboratory, bioinformatics and statistical analyses.
