# Supplementary material for: Fungal diversity and seasonal succession in ash leaves infected by the invasive ascomycete Hymenoscyphus fraxineus
Source: New Phytol. 2016 Sep 26;213(3):1405–17. doi: 10.1111/nph.14204 (PMC5347882; doi:10.1111/nph.14204)

New Phytologist

***New Phytologist* Supporting information**

Article title: **Fungal diversity and seasonal succession in ash leaves infected by the invasive ascomycete *Hymenoscyphus fraxineus***

Authors: Hugh Cross, Jørn Henrik Sønstebø, Nina E. Nagy, Volkmar Timmermann, Halvor Solheim, Isabella Børja, Håvard Kauserud, Tor Carlsen, Barbara Rzepka, Katarzyna Wasak, Adam Vivian-Smith and Ari M. Hietala

Article acceptance date: 15 August 2016

The following Supporting Information is available for this article:

Methods S1 Detailed laboratory, bioinformatics and statistical analyses.
Fig. S1 Monthly precipitation and mean temperature in 2011 and 2012.
Fig. S2 Damage assessment of sampled ash trees in early (22.6.) and late (23.8.) summer 2012.
Fig. S3 *Hymenoscyphus fraxineus* DNA amount in tissues (ng DNA per milligram tissue) collected from healthy and diseased ash trees throughout the summer 2012 and analyzed by a qPCR assay specific to the DNA of the fungus.
Fig. S4 Alpha rarefaction curves of individual samples for next generation sequencing ITS-1 (A) and ITS-2 (B) datasets.
Fig. S5 Seasonal changes in ITS-1 and ITS-2 read proportions of fungal taxa at the experimental stand.
Table S1 The most common fungal genera present in both ITS-1 and -2 datasets, and their sequence proportions (%) in leaf tissues in early (29.06.–11.07.), mid and late summer (12.08.–25.08.) in 2011.
Table S2 The most common fungal genera detected in one ITS dataset only and their sequence proportions (%) in leaf tissues in early (29.06.–11.07.), mid (18.07.–02.08) and late summer (12.08.–25.08.) in 2011.
Table S3 ITS-1 sequence percentages of fungi detected in air samples captured by a volumetric spore sampler at the experimental ash stand in 2010.
Table S4 ITS-2 sequence percentages of fungi detected in air samples captured by a volumetric spore sampler at the experimental ash stand in 2010.

Table S5 Pearson product moment correlation between ITS-2 sequence proportions of *Hymenoscyphus fraxineus* and the most common fungal general in different ash leaf tissues across the season 2011.

Fig. S6 Portion of nucleotide alignment of *Hymenoscyphus* ITS-2 sequences including a putative new undescribed genotype of *H. fraxineus.*

Fig. S7 Seasonal changes in *H. fraxineus* DNA amount, determined by qPCR and by ITS-2 read percentages of the two main sequence variants assigned to this species.

Methods S1 Detailed laboratory, bioinformatics and statistical analyses

Real-time PCR

The real-time PCR detection of *H. fraxineus* DNA was performed using FAST BLUE qPCR MasterMix Plus w/o UNG for probe Assay Low ROX (Eurogentec, Seraing, Belgium), and the ITS-1 region specific forward primer Cfrax-F (5′-ATT ATA TTG TTG CTT TAG CAG GTC-3’), the reverse primer Cfrax-R (5′-TCC TCT AGC AGG CAC AGT C-3′) and the probe Cfrax-P (5′-FAM-CTC TGG GCG TCG GCC TCG-BHQ1-3′) described and tested for species specificity by Ioos *et al.* (2009). The primer and probe concentrations per assay were 300 nM and 100 nM, respectively (Ioos *et al.,* 2009).

A standard curve with known concentrations of *H. fraxineus* DNA was prepared from pure cultures as previously described (Hietala *et al.,* 2013). To ensure that the cycle threshold values from the experimental samples were within the standard curves, and that PCR inhibitory compounds potentially present in undiluted samples remained low in the assay, a 3-log dilution series was prepared so that for each sample the undiluted DNA and the 10- and 100-fold dilutions were used as templates for real-time PCR. Both the experimental and standard curve samples comprised 3 μl of DNA as the template in 25-μl reactions. The duplicates prepared for each sample were heated at 95°C for 10 min, followed by 40 cycles of 95°C for 15 s and 65°C for 55 s. Fluorescence emissions were detected with an ABI Prism 7700 (Applied Biosystems)

Preparation of ITS-1 NGS libraries

This entailed a nested PCR approach, using the fungal-specific primers ITS1F and ITS4 (White *et al*., 1990; Gardes & Bruns, 1993) in the first step and fusion primers ITS5 and ITS2 (White *et al*., 1990) in the nested step, using 50× diluted product from the first PCR. Fusion primers contained 16 different 10 bp unique tags and 454 pyrosequencing adaptors A and B to both ITS5 and ITS2, respectively. PCR was performed in 20 μl reaction volumes containing 2 μl template DNA and 18 μl reaction mix. Final

concentrations in the PCR were 0.10 mM dNTP mix, 0.125 µM of each primer and 0.5 units Finnzymes Phusion Hot Start II polymerase (Thermo Fisher Scientific, Massachussets, USA). The amplification program for both PCRs was: 30 s at 98ºC, followed by 20 cycles of 10 s at 98ºC, 20 s at 50ºC, 20 s at 72ºC and a final extension step at 72ºC for 7 min before storage at -20ºC. PCR products were normalized to a single DNA concentration using the SequelPrep Normalization Plate (Invitrogen, CA, USA) and then cleaned with Wizard_SV PCR Clean-Up System (Promega, Madison, WI, USA). GS FLX sequencing of the tagged amplicons was performed at the Norwegian High-Throughput Sequencing Centre (http://www.sequencing.uio.no) using one 454 plate divided into eight compartments. We included two negative controls through all analyses from the DNA extraction step.

Processing of ITS-2 NGS libraries

The fungal ITS-2 region was amplified using the degenerate gITS7 primer (Ihrmark *et al.*, 2012) together with the ITS4 primer (White *et al.*, 1990). Reactions comprised 45 µl of Platinum PCR SuperMix High Fidelity (ThermoFisher), 0.49 µM gITS7 primer, 0.29 µM ITS4 primer and 2 µl of DNA sample in a total volume of 51 µl. PCR reactions were conducted using the following cycling conditions; 94ºC for 2 min, followed by 35 cycles of 94ºC for 30 s, 56ºC for 30 s, and 68ºC for 60 s. Each reaction was cleaned with 1.1 volumes of Ampure XP (Beckman Coulter, Inc, Pasadena), and the products were ligated to barcoded adaptors as outlined in the Ion Amplicon Library Preparation user guide and Ion Xpress fragment kit (PN 4468326 rev B and P/N 4471252, respectively), and where the A adaptor contained a sample specific Ion Xpress_barcode identification sequence (Thermo Fisher Scientific, Massachusetts, USA; catalogue number 4471250). The resulting products were pooled in equal amounts and purified using another round of Ampure XP cleaning, and analyzed on a Bioanalyzer 2100 High Sensitivity DNA Chip (Agilent Biosciences). Subsequently library pools were diluted and sequenced according to Ion Torrent manufacturer specifications (Thermo Fisher Scientific, Massachusetts, USA) on the Ion PGM using 314 v2 chips with 400 bp chemistry. Sequences were inspected using FastQC (Andrews, 2011).

Processing of raw sequences

All raw fastq reads from 454 and Ion Torrent were processed and filtered using the programs Cutadapt (Martin, 2011) and FastX-Toolkit (http://hannonlab.cshl.edu/fastx_toolkit/index.html). First, all reads were sorted by barcode (MID) sequence tag into separate files using the fastx_barcode_splitter.pl script in FastX-Toolkit. A bash script was used to process all files in turn, using the following steps: the primer from the 5` end of each read was trimmed using Cutadapt, then all trimmed reads were reverse complemented using FastX, and the primer was again trimmed from the 5` end. The reads were then filtered for quality using FastX, keeping a minimum of 90% of sequences with a phred score of 20, renamed using a custom python script, and then FastX was used to convert the files to FASTA format.

Initial clustering of NGS reads

The overall method for clustering reads and assigning taxonomy followed a modified and customized version of open-reference OTU clustering, as described by Rideout *et al.* (2014), as this method has been found to be a good compromise between OTU stability and the inclusion of novel taxa (He *et al.*, 2015). All processed reads were dereplicated using USEARCH version 8 (Edgar, *et al.,* 2011). The program Swarm (Mahe *et al*., 2014) was used to cluster reads into Operational Taxonomic Units (OTUs), using the local clustering threshold of 1. OTUs represented by only one sequence and chimeric sequences were removed using USEARCH and the UNITE-INSD fungal ITS database (Dynamic version 7, 02-03-2015 release) (http://unite/ut/ee/repository.php).

The remaining OTUs were used as queries for searches using the BLAST+ program, first against the UNITE database, and then remaining OTUs that had no hit to UNITE were searched against the NCBI nt database, removing any results that matched nondescript sequences above family level (e.g. 'uncultured', 'fungal endophyte', 'Ascomycota sp.', 'Basidiomycota sp', 'Sordariomycete sp.'). Before submitting for BLAST search, the ribosomal portions of all OTU sequences were removed using the ITSx program (Bengtsson-Palme *et al*., 2013). This was done to reduce the number of spurious hits caused by matches in the SSU, 5.8S, and LSU portions of the sequence. All reference sequences from these two BLAST searches with a qcov score greater than 60 were then downloaded and assembled into a fasta file.

Additional ITS sequences of Norwegian strains of *Hymenoscyphus fraxineus* and *H. albidus* were added to the reference sequence file. This reference fasta file was then used as a first stage for matching the reads to a reference. All reads were first clustered with the reference ITS sequences ('closed reference OTU picking') using the USEARCH usearch_global option at a threshold ID of 97%. Any reads that did not cluster with any UNITE or NCBI sequence (within the threshold) were extracted from the results file (usearch cluster, or '.uc' file), and then clustered into de Novo OTUs using USEARCH ('open reference OTU picking'). The results from these two clusters procedures (excluding reads without any hit) were then converted to tables using the USEARCH python script 'uc2otutab.py', and combined into a single table.

Taxonomy assignment

For the closed reference OTU clustering, any reads that matched to a UNITE or NCBI species was assigned to that species (using the 97% cutoff). The OTUs that were matched to reads in the second clustering step were assigned taxonomy using both the Qiime (Caporoso *et al*., 2010) method (RDP algorithm) and the USEARCH utax algorithm, to the UNITE sequence database. The results of the utax method assign a *P*-value to each taxonomic rank, indicating the probability of the assignment at that level being correct. As of this writing, this technique has not been thoroughly tested, though it compares favorably to the RDP method (see

http://www.drive5.com/usearch/manual/utax_vs_rdpc_its.html) at higher levels of resolution (http://drive5.com/usearch/manual/utax_vs_rdpc_its.html); in our results the utax results were not significantly different from the Qiime assignments. The results were converted to a Qiime-compatible format using a custom Python script, setting a minimum *P*-value of 95 for taxonomic rank except kingdom, which was set to 100. At each rank, if the threshold fell below the p value, the next rank below was assigned; thus the highest resolution rank assigned was the highest rank above the threshold, unless the highest rank was Kingdom, in which case the assignment was registered as 'Unassigned'. The highest rank assigned with the utax method was to genus.

For the NCBI reference sequences, the taxonomic lineage was taken from the NCBI Taxid file. These were also converted to qiime format. The UNITE reference taxonomy was obtained from the pre-formatted Qiime file of the same version as the BLAST search. These taxonomies, together with the OTU taxonomy, were combined for downstream analyses using Qiime.

Statistical and taxonomic analyses

Abundance tables from the USEARCH read clustering were converted to the biom format using the software BIOM (McDonald *et al*., 2012), and then sample and taxonomic metadata were added to the final biom tables. Qiime (Caporoso *et al*., 2010) was then used to summarize taxonomic tables and plot relative abundance of taxa across all samples, and by date and tissue type. The biom files were imported into the program Megan version 5.10.3 (Huson *et al*., 2011), and this program was used to map and chart the taxonomy, compare the ITS-1 and ITS-2 results, and calculate Alpha Rarefaction curves. R was used to plot the alpha rarefaction results. To examine relationships between samples, normalized abundance tables were created using the CSS algorithm (Paulson *et al*., 2013) as implemented with a Qiime script and then Principal Components Analysis (PCA) was conducted using the R package vegan (Oksanen *et al*., 2015).

In order to get a more precise estimate of the relative representation of different *Hymenoscyphus* species and genotypes, all reads that mapped to *Hymenoscyphus* in the USEARCH global search (either directly to UNITE sequence or OTU) were extracted and then remapped to a single *Hymenoscyphus fraxineus* representative sequence using the BWA mem algorithm (Li & Durbin, 2009). For this search the parameters used were all default except that the gap extension penalty was raised in order to favor better alignment over single base mismatches. Then each read was reassigned to different *Hymenoscyphus* species/genotypes based on key characters at different positions of the aligned sequence. Additionally, reads assigned to other taxa of relatively high abundance (greater than 1–2%) were extracted and compared to reference sequences and OTUs to determine their relative species composition and diversity. These reads and/or references were aligned using the Geneious software package Version 6 (Kearse *et al*., 2012) for comparison.

Non-linear modelling of seasonal atmospheric ascospore density

Our previous study (Hietala *et al.*, 2013) reported *H. fraxineus* ascospore density at the experimental stand in the period 2009-2011. A four-parameter Weibull Probability Distribution Model was fitted with Sigmaplot v12.5 (Systat Software Inc.) to log- transformed ascospore density data. A best fit was derived with an r^2 value of 0.681. With back transformation this provided an *x*-intercept of 174 ± 6 d and a peak of ascospore saturation at 212 d. A 95% prediction interval was also determined for the fitted curve using the back transformed data (Fig. 2c).

References

Bengtsson-Palme J, Ryberg M, Hartmann M, Branco S, Wang Z, Godhe A, Wit P, Sánchez-García M, Ebersberger I, Sousa F. **2013**. Improved software detection and extraction of ITS1 and ITS2 from ribosomal ITS sequences of fungi and other eukaryotes for analysis of environmental sequencing data. *Methods in Ecology and Evolution* **4**: 914–919.

Caporaso JG, Kuczynski J, Stombaugh J, Bittinger K, Bushman FD, Costello EK, Fierer N, Pena AG, Goodrich JK, Gordon JI. **2010**. QIIME allows analysis of high-throughput community sequencing data. *Nature Methods* **7**: 335–336.

Edgar RC. **2010**. Search and clustering orders of magnitude faster than BLAST. *Bioinformatics* **26**: 2460–2461.

He Y, Caporaso JG, Jiang X-T, Sheng H-F, Huse SM, Rideout JR, Edgar RC, Kopylova E, Walters WA, Knight R *et al.* **2015**. Stability of operational taxonomic units: an important but neglected property for analyzing microbial diversity. *Microbiome* **3**: 20.

Hietala AM, Timmermann V, Børja I, Solheim H. **2013**. The invasive ash dieback pathogen *Hymenoscyphus pseudoalbidus* exerts maximal infection pressure prior to the onset of host leaf senescence. *Fungal Ecology* **6**: 302–308.

Huson DH, Mitra S, Ruscheweyh H-J, Weber N, Schuster SC. **2011**. Integrative analysis of environmental sequences using MEGAN4. *Genome Research* **21**: 1552–1560.

Kearse M, Moir R, Wilson A, Stones-Havas S, Cheung M, Sturrock S, Buxton S, Cooper A, Markowitz S, Duran C *et al.* **2012**. Geneious Basic: an integrated and extendable desktop software platform for the organization and analysis of sequence data. *Bioinformatics* **28**: 1647–1649.

Li H, Durbin R. **2009**. Fast and accurate short read alignment with Burrows–Wheeler transform. *Bioinformatics* **25**: 1754–1760.

Mahé F, Rognes T, Quince C, de Vargas C, Dunthorn M. **2015**. *Swarm v2: highly-scalable and high-resolution amplicon clustering. PeerJ PrePrints* **3**: e1503.

Martin M. **2011**. Cutadapt removes adapter sequences from high-throughput sequencing reads. *EMBnet. Journal* **17**: 10–12.

McDonald D, Clemente JC, Kuczynski J, Rideout JR, Stombaugh J, Wendel D, Wilke A, Huse S, Hufnagle J, Meyer F. **2012**. The Biological Observation Matrix (BIOM) format or: how I learned to stop worrying and love the ome-ome. *GigaScience* **1**: 7.

Oksanen J, Blanchet FG, Kindt R, Legendre P, Minchin PR, O'Hara RB, Simpson GL, Solymos P, Stevens MHH, Wagner H. **2015**. *vegan: Community Ecology Package*.

Paulson JN, Stine OC, Bravo HC, Pop M. **2013**. Differential abundance analysis for microbial marker-gene surveys. *Nature Methods* **10**: 1200–1202.

Rideout JR, He Y, Navas-Molina JA, Walters WA, Ursell LK, Gibbons SM, Chase J, McDonald D, Gonzalez A, Robbins-Pianka A *et al.* 2014. Subsampled open-reference clustering creates consistent, comprehensive OTU definitions and scales to billions of sequences. *PeerJ* **2**: e545.

Fig. S1 Monthly precipitation and mean temperature in 2011 and 2012 in relation to long-term means 1961-1990 at a meteorological station located on a field *c*. 2000 m away from the studied ash forest.

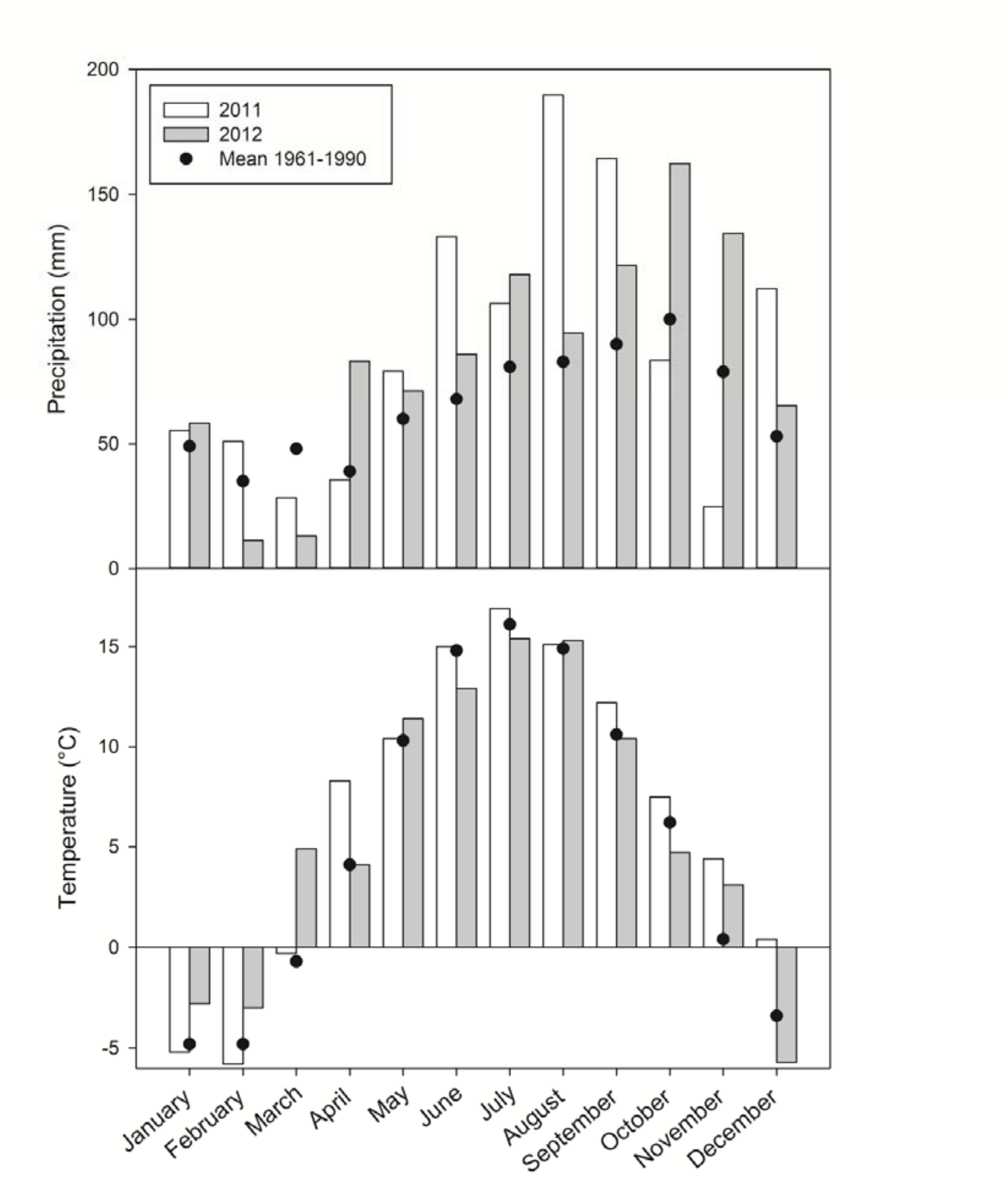

Fig. S2 Damage assessment of sampled ash trees in early (22.6.) and late (23.8.) summer 2012. Parameters assessed: Defoliation, dieback, epicormic shoots, dry or discolored leaves and dead tops. Score based on combined damage classes (defoliation: 0 = no damage, 1 = 1-10 % defoliation, 2 = 11-25 %, 3 = 26-50 %, 4 = 51-75 %, 5 = 76-90 %, 6 = 91-99 %, 7 = 100 % defoliation, 8 = dead; other parameters: 0 = none, 1 = slight, 2 = moderate, 3 = severe).

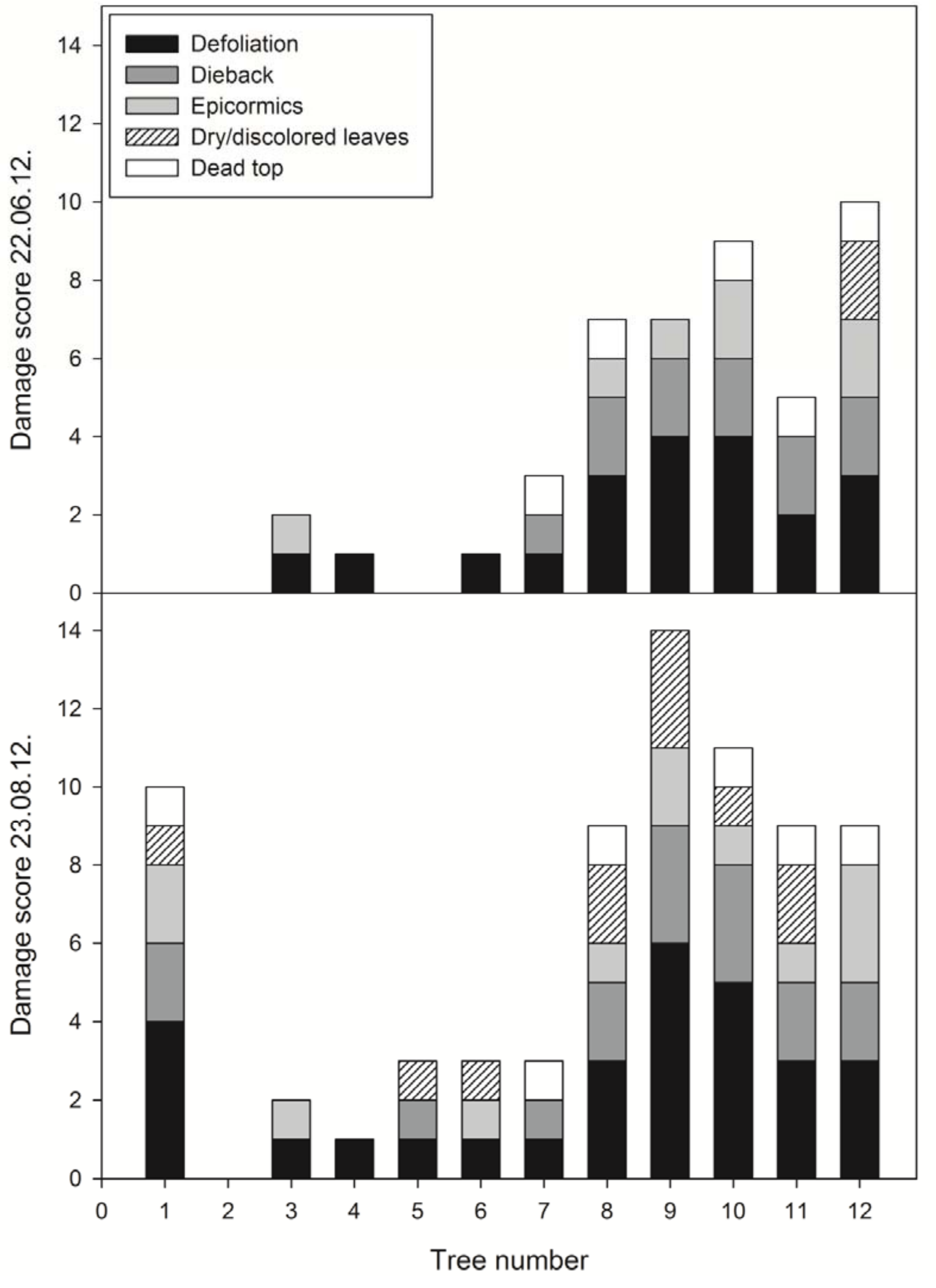

Fig. S3 *Hymenoscyphus fraxineus* DNA amount in tissues (ng DNA per milligram tissue) collected from healthy and diseased ash trees throughout the summer 2012 and analyzed by a qPCR assay specific to the DNA of the fungus. The trees were grouped as healthy (trees # 2, 3, 4, 5, 6, 7) or diseased (trees #1, 8, 9, 10, 11, 12) based on their condition at the end of the growing season (see Fig. S2). The values represent means of two trees per time point, while the error bars represent standard deviation.

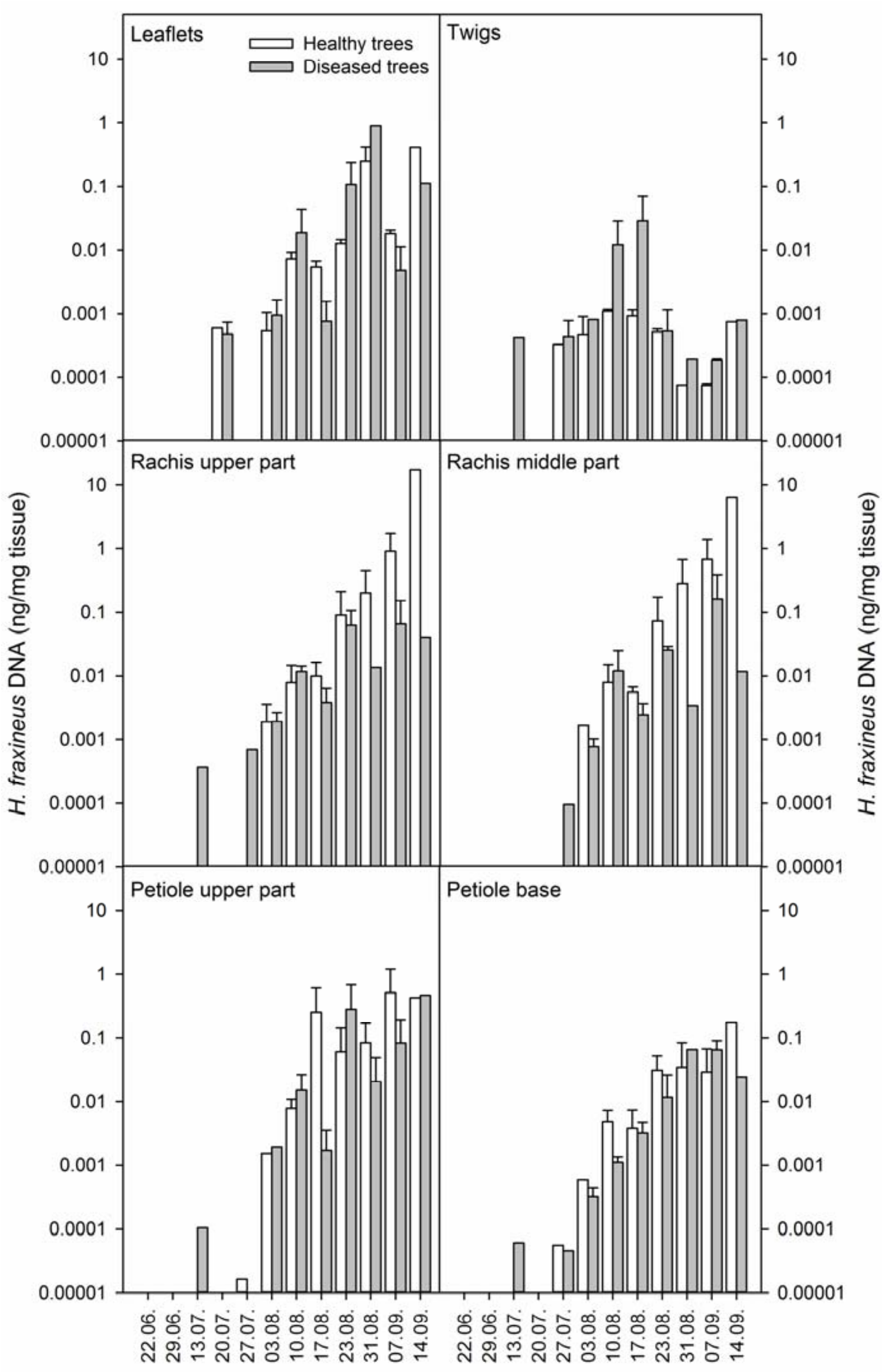

Fig S4 Alpha rarefaction curves of individual samples for next generation sequencing ITS-1 (a) and ITS-2 (b) datasets.

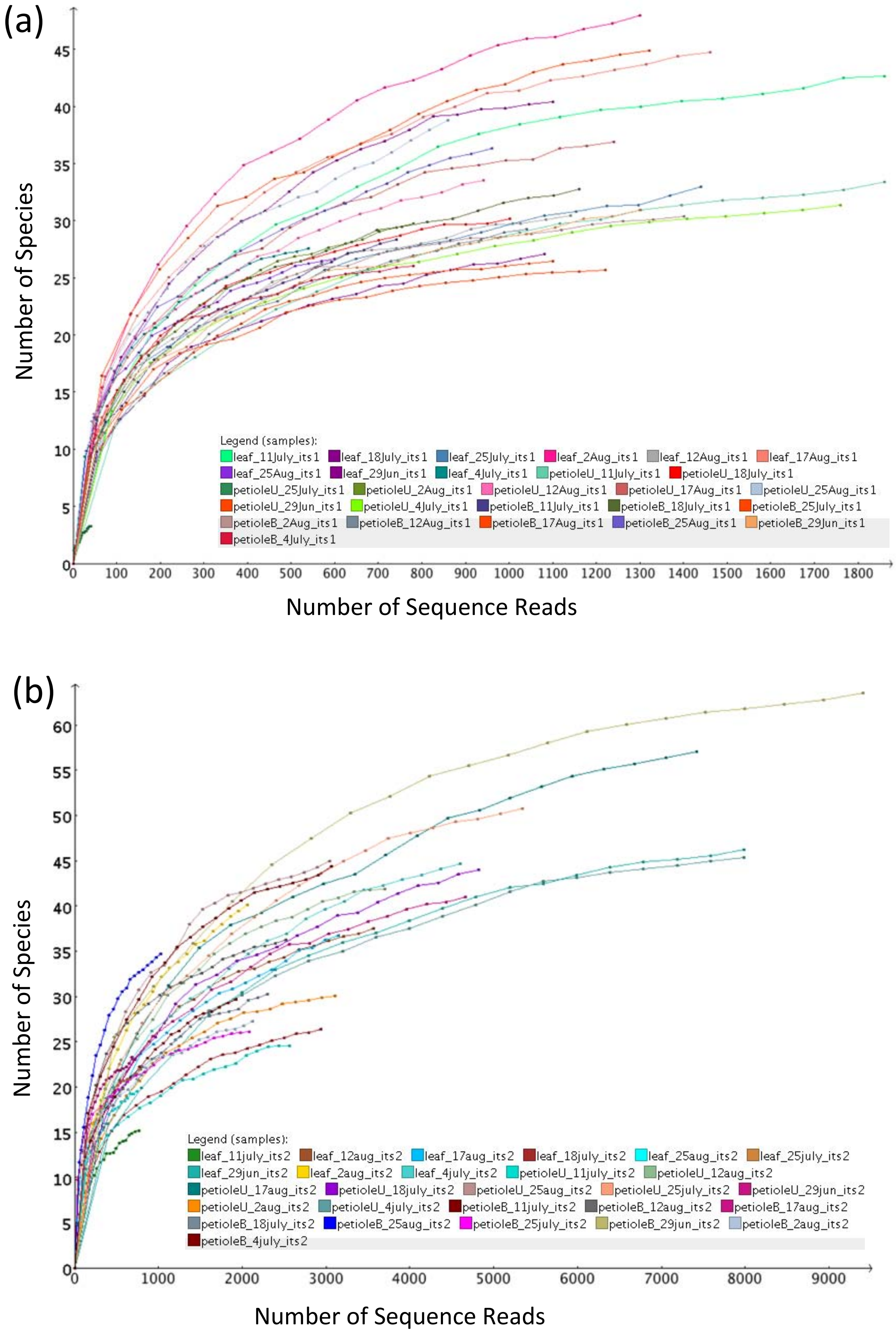

Fig. S5 Seasonal changes in ITS-1 and ITS-2 read proportions of fungal taxa at the experimental stand. Dominant fungal orders (A, basidiomyectous yeasts; B, ascomycetous and basidiomycetous biotrophs; C, ascomycetous endophytes) associated with ash leaf tissues in season 2011. (D–F) The proportion of these groups in volumetric air samples collected by Burkard spore sampler – the air samples analyzed represent 24-h samples collected from midnight to midnight during the given day at the experimental stand in 2010.

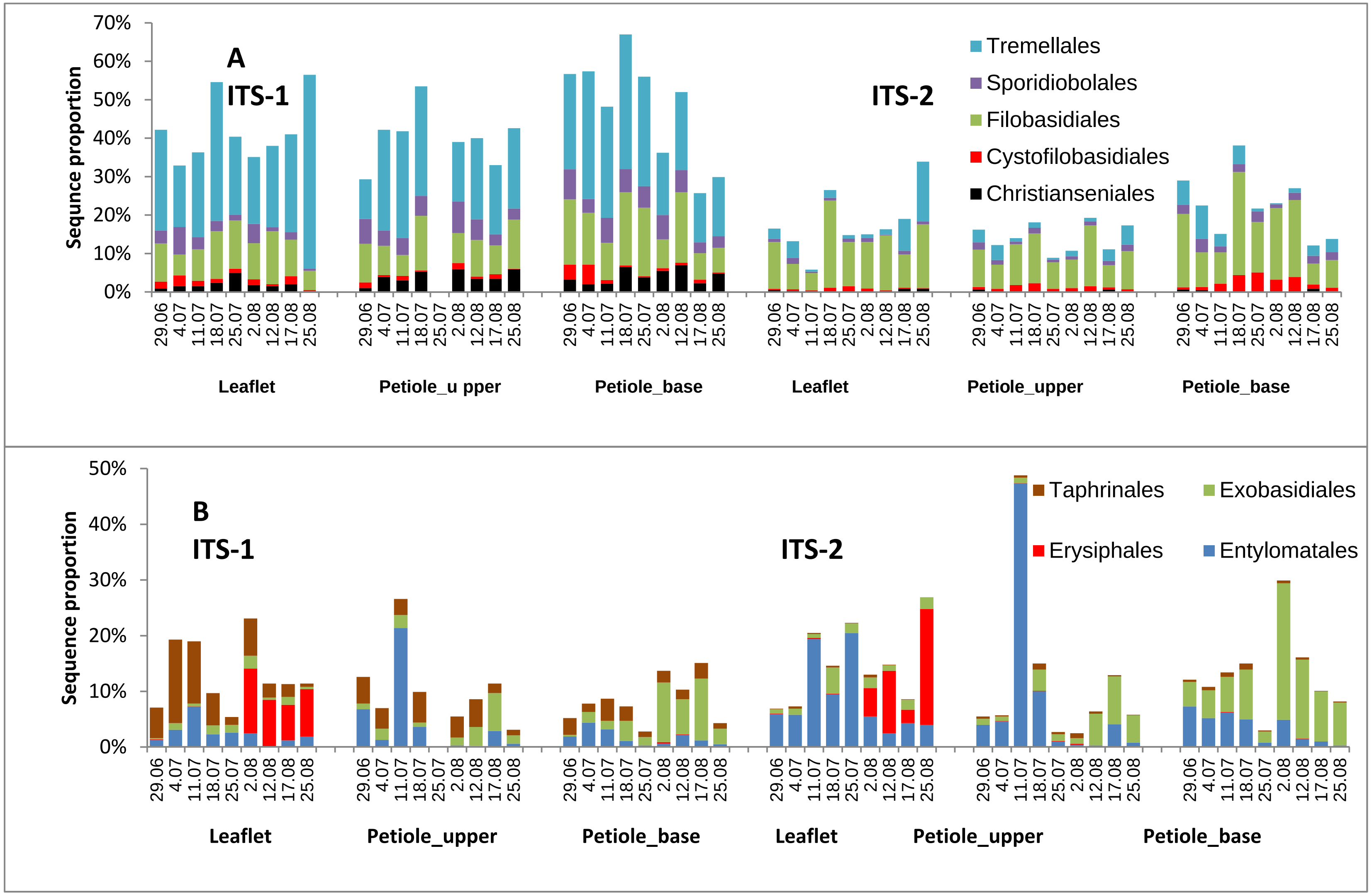

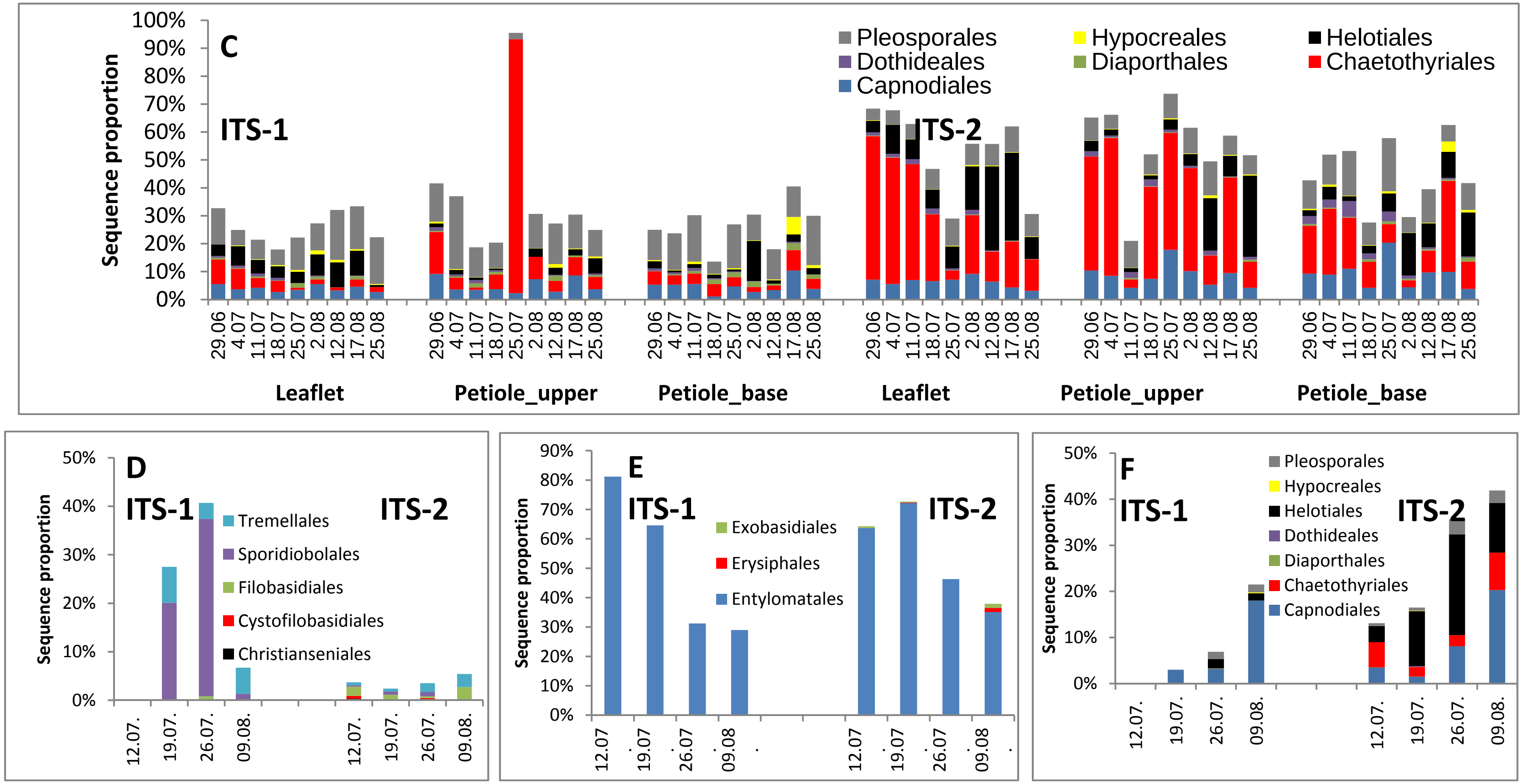
C
ITS-1
ITS-2
Sequence proportion
100%
90%
80%
70%
60%
50%
40%
30%
20%
10%
0%
Pleosporales
Dothideales
Capnodiales
Hypocreales
Diaporthales
Helotiales
Chaetothyriales
29.06
4.07
11.07
18.07
25.07
2.08
12.08
17.08
25.08
Leaflet
Petiole_upper
Petiole_base
Leaflet
Petiole_upper
Petiole_base
D
ITS-1
ITS-2
Sequence proportion
Tremellales
Sporidiobolales
Filobasidiales
Cystofilobasidiales
Christianseniales
12.07.
19.07.
26.07.
09.08.
E
ITS-1
ITS-2
Sequence proportion
Exobasidiales
Erysiphales
Entylomatales
12.07
19.07
26.07
09.08
F
ITS-1
ITS-2
Sequence proportion
Pleosporales
Hypocreales
Helotiales
Dothideales
Diaporthales
Chaetothyriales
Capnodiales
12.07.
19.07.
26.07.
09.08.

Table S1 The most common fungal genera present in both ITS-1 and -2 datasets, and their sequence proportions (%) in leaf tissues in early (29.06.–11.07.), mid (18.07.–02.08.9) and late summer (12.08.–25.08.) in 2011. For genera which showed statistically significant differences within a tissue type between the three sampling periods (LSD post hoc test), means that differ from each other ($P < 0.05$) are indicated with different letters. Correlation coefficient indicates product moment correlation between the ITS-1 and -2 sequence proportions for a given genus (**, $P < 0.01$; *, $P < 0.05$).

Genus	Putative	ITS	Leaflet			Petiole upper			Petiole base			Correlation
	Mode	Region	29.06-11.07.	18.07.-02.08.	12.08.-25.08.	29.06-11.07.	18.07.-02.08.	12.08.-25.08.	29.06-11.07.	18.07.-02.08.	12.08.-25.08.	coefficient
Bullera	Ep[#]	1	0.1a	0.3a	8.3b	0.1[§]	0.5	1.2	0.5	0.1[§]	0.5	0.99**
		2	0.0a	0.0a	3.7b	0	0.3	0.3	0.7	0.1	0.2	
Cryptococcus	Ep	1	21.4	25.9	22.1	26.7	19	38.5	29.6	32.6	21.4	0.61**
		2	3.6	4.7	8.1	5.8	4.3	7.7	7.7	9.2	6.7	
Dioszegia	Ep	1	5	4.9	5.7	2.8	2.6	4.8	7.3	5.4	3.9	0.56**
		2	1.7	1.2	3.6	1.6	0.6	1.7	3.7	1.7	1.6	
Rhodotorula	Ep	1	0.3	0.4	0.3	1.4ab	1.9a	2.3b	1	2.1	2.2	0.53**
		2	0.3	0.4	0.3	0.6	0.6	0.7	1.2	1.2	1.2	
Sporobolomyces	Ep	1	1.4	1.4	0.5	1.8	1.2	2.3	2.0ab	3.7a	0.9b	0.27
		2	0.4	0.2	0.2	0.5ab	0.2a	0.5b	1.1	0.6	0.7	
Tilletiopsis	Ep	1	3.9	2.3	0.8	10.0a	1.2b	1.4b	2.9a	0.7ab	0.2b	0.92**
		2	10	10.6	3.4	17.4a	3.5a	1.7b	5.6a	3.2a	0.5b	
Aureobasidium	Ep	1	0.7	0.4	0.0[§]	0.9	0.2	0.4	0.7	0.3	0.2	0.57**
		2	1.3a	1.1a	0.1b	1.4	1	0.8	3.3a	1.8a	0.5b	
Cladophialophora	Ep	1	5.9	1.6	1.2	6.3	3.9	4	2.3	0.9	1.5	0.41*
		2	43.6	14.6	10.9	28.9	35.7	15	17.5	5	13.3	
Entyloma	Op	1	0	0.1	0.3	0.0[§]	0.1[§]	0.3	0.1a	0.0[§]	1.1b	-0.11
		2	1	2.1	0.3	2.5	0.5	0.1	0.9	0.6	0.5	
Exobasidium	Op	1	0.6a	1.7b	0.7a	1.2	0.7	5.8	1.8a	5.3ab	6.7b	0.84**
		2	0.9a	3.0b	1.7ab	1.2a	2.1a	6.6b	5.4	12.1	11.2	
Phyllactinia	Op	1	0.0[§]	3.8	7.8	ND^	ND	0.0[§]	ND	ND	ND	0.77**
		2	0.1a	1.7a	11.5b	0.1	0.1	0	0	0	0	
Taphrina	Op	1	6.8a	2.3b	0.8b	1.8ab	2.4a	1.2b	1.6	0.7	0.8	0.14
		2	0.2	0.3	0.1	0.4	0.8	0.3	0.6a	0.9a	0.3b	

Epicoccum	En/P	1	0.1a	0.2ab	1.3b	0.1	0.0[§]	1.9	0.1a	0.1ab	0.4b	-0.31
		2	0.1[§]	0.4	0.2	0.2[§]	0.4	0.2[§]	0.1[§]	0.3	0.3	
Fusarium	En/P	1	0.2a	0.5ab	0.6b	0.2	0.1	0.9	0.4ab	0.3a	2.4b	0.96**
		2	0.1	0.2	0.2	0.1	0.2	0.4	0.4	0.3	1.3	
Hymenoscyphus	En/P	1	0	0	0.0[§]	0	0	0.1	0	0	0.0[§]	0.49**
		2	0.3a	2.6b	18.0c	0.3a	0.9a	15.5b	0.2a	1.2b	8.9c	
Leptosphaeria	En/P	1	0.2	0.2	0.1	0.1	0.1[§]	0.2	0.9	0.9	0.7	0.39*
		2	0.2	0.6	0.2	0.5	0.5	0.4	1.3	0.8	0.8	
Naevala	En/P	1	3.7	2.9	2.4	0.3	0.1	0.4	0.4	0.2	0.1	0.66**
		2	1.3	0.1	0.6	0.2	0	0.1	0.3	0	0	
Phoma	En/P	1	3.9a	3.5a	8.9b	5.2	2.7	7.2	5.5	3.2	3.9	0.44*
		2	0.1ab	0.1a	0.8b	0.2	0.1	0.9	0.2	0	0.7	
Devriesia	En/P	1	0.1	0.3	0.2	0.1	0.8	0.5	0.1[§]	0.3	0.8	0.35
		2	0.1a	0.3b	0.2a	0.1a	0.5b	0.5b	0.2	1.5	0.7	

[#]Putative feeding mode based on literature: Ep, epiphyte; Op, obligatory parasite; En/P endophyte or facultative parasite.

[§]Detected in the tissue only in one sample within the given sampling period.

^ND, not detected in the tissue within the given sampling period.

Table S2 The most common fungal genera detected in one ITS dataset only and their sequence proportions (%) in leaf tissues in early (29.06.–11.07.), mid (18.07.–02.08) and late summer (12.08.–25.08.) in 2011. For genera which showed statistically significant differences within a tissue type between the three sampling periods (LSD post hoc test), means that differ from each other (*P* < 0.05) are indicated with different letters.

Genus	Putative Mode	ITS region	Leaflet 29.06-11.07.	Leaflet 18.07.-02.08.	Leaflet 12.08.-25.08.	Petiole upper 29.06-11.07.	Petiole upper 18.07.-02.08.	Petiole upper 12.08.-25.08.	Petiole base 29.06-11.07.	Petiole base 18.07.-02.08.	Petiole base 12.08.-25.08.
Bensingtonia	Ep#	1	0.2	0.3	0.8	0.6	0.7	1.5	0.3	0.7	1.2
Lalaria	Ep	1	1.5a	1.0a	0.2b	1.6	0.5	2.1	1.2	1	0.7
Udeniomyces	Ep	1	0.4	0.4	0.5	0.6	0.0§	0.2	1.6	0.1	0.1§
Ceramothyrium	Ep	2	1	0.4	0.5	0.4a	0.1b	0.2a	0.2	0.2	0.1
Boeremia	En/P	1	0.6a	0.6a	3.4b	0.6	0.7	1.6	1.3a	0.3b	1.2a
Cryptosporiopsis	En/P	1	ND^	0.0§	0	ND	0.1§	0	0.0§	4.4	0
Diaporthe	En/P	1	0.2	0.3	0.1	0.1a	0.0§	1.1b	0.2a	0.5ab	0.8b
Gyoerffyella	En/P	1	1.4	1.4	0.8	0.1	0.1	0.2	0.2	0.3	0.1
Phomopsis	En/P	1	0.2	0.7	0.3	0.5	0.2§	0.2	0.4	1.1	0.6
Acicuseptoria	En/P	2	0.1§	0.4	0	0.1§	0.6	0.5	0.5	0.2	0.5
Cladosporium	En/P	2	0.3	0.1	0.4	0.4	0.1	0.4	0.4a	0.1b	0.3ab
Knufia	En/P	2	0.0a	0.1a	0.5b	0.03a	0.0a	0.8b	0	0.4	0.3
Paraleptosphaeria	En/P	2	0.6	0.7	0.1	1.5	0.9	1.1	3.0a	0.3b	1.3a
Peyronellaea	En/P	2	2.2	5.2	3.8	3.3	4.5	3.5	4.4	5	3.4
Pleospora	En/P	2	0.7a	ND^	1.3b	0.7	ND	0.9	1.1	ND	0.9
Phallus	S	1	0.0§	1.4	0.7	0.0§	1.7	5.3	ND	0.1a	3.9b

#Putative feeding mode based on literature: Ep, epiphyte; En/P endophyte or facultative parasite; S, saprophyte.
§Detected in the tissue only in one sample within the given sampling period.
^ND, not detected in the tissue within the given sampling period.
#

Table S3 ITS-1 sequence percentages of fungi detected in air samples captured by a volumetric spore sampler at the experimental ash stand in 2010. Absence of data indicates that the given genus was not detected in the sample.

Systematic position	Genus	Mode*	Date			
			12.07.	19.07.	26.07.	09.08.
p__Basidiomycota;c__Microbotryomycetes;o__Sporidiobolales;f__Incertae sedis	Sporobolomyces	Ep		14.0	29.0	1.2
p__Basidiomycota;c__Tremellomycetes;o__Tremellales;	Bullera, Dioszegia, Hannaella	Ep		7.5	3.3	6.5
p__Basidiomycota;c__Tremellomycetes;o__Tremellales;f__unidentified	unidentified	Ep			0.4	
p__Basidiomycota;Exobasidiomycetes;o__Entylomatales;f__Entylomataceae	Tilletiopsis	Ep	81.3	64.5	30.0	14.6
	SUM		81.3	71.9	33.7	21.1
p__Basidiomycota;c__Exobasidiomycetes;o__Entylomatales;Other	unidentified	Op		0.2	1.2	13.8
p__Basidiomycota;c__Exobasidiomycetes;o__Entylomatales;f__Entylomataceae	Entyloma	Op				0.7
p__Basidiomycota;c__Exobasidiomycetes;o__Exobasidiales;Other	unidentified	Op				0.7
	SUM			0.2	1.2	15.3
p__Ascomycota;c__Dothideomycetes;o__Pleosporales;f__Phaeosphaeriaceae	Leptospora	En/P			0.4	0.3
p__Ascomycota;c__Dothideomycetes;o__Pleosporales;f__unidentified	unidentified	En/P				
p__Ascomycota;c__Dothideomycetes;o__Capnodiales;f__Davidiellaceae	Cladosporium	En/P		1.4	0.1	4.3
p__Ascomycota;c__Eurotiomycetes;o__Eurotiales;f__Trichocomaceae	unidentified	En/P			0.4	
p__Ascomycota;c__Leotiomycetes;o__Helotiales;f__Incertae_sedis	Xenopolyscytalum	En/P			0.2	
p__Ascomycota;c__Leotiomycetes;o__Helotiales;f__Helotiaceae	Hymenoscyphus	En/P			0.4	
p__Ascomycota;c__Sordariomycetes;o__Sordariales;f__Lasiosphaeriaceae	Lasiosphaeris	En/P				1.2
p__Ascomycota;c__Sordariomycetes;o__Incertae_sedis;f__Plectosphaerellaceae	Plectosphaerella	En/P				3.1
p__Ascomycota;c__Sordariomycetes;o__Hypocreales;f__Nectriaceae	Nectria	En/P				0.2
p__Ascomycota;c__Sordariomycetes;o__Diaporthales;f__Gnomoniaceae	Gnomonia	En/P			0.1	
	SUM			1.4	1.7	9.1
p__Basidiomycota;c__Agaricomycetes;o__Cantharellales;f__Hydnaceae	Sistotrema	S	16.7		1.1	
p__Basidiomycota;c__Agaricomycetes;o__Russulales;f__Peniophoraceae	Peniophora	S		0.4	1.7	6.1
p__Basidiomycota;c__Agaricomycetes;o__Polyporales;f__Polyporaceae	Polyporus	S		2.4		0.1
p__Basidiomycota;c__Agaricomycetes;o__Atheliales;f__Atheliaceae	Tylospora	S	2.1		0.2	0.4
p__Basidiomycota;c__Agaricomycetes;o__Agaricales;f__Inocybaceae	Crepidotus	S				0.9
p__Basidiomycota;c__Agaricomycetes;o__Polyporales;f__Ganodermataceae	Ganoderma	S			0.8	
p__Basidiomycota;c__Agaricomycetes;o__Hymenochaetales;f__Schizoporaceae	Hyphodontia	S				0.7
p__Basidiomycota;c__Agaricomycetes;o__Russulales;f__Bondarzewiaceae	Heterobasidion	S		0.1	0.4	
p__Basidiomycota;c__Agaricomycetes;o__Agaricales;f__Marasmiaceae	Calyptella	S		0.4		

p__Basidiomycota;c__Agaricomycetes;o__Agaricales;f__Psathyrellaceae	Coprinopsis	S				0.4
p__Basidiomycota;c__Agaricomycetes;o__Agaricales;f__Cortinariaceae	Cortinarius	S		0.4		
	SUM		18.8	3.6	4.1	8.6

*Putative feeding mode based on literature: Ep, epiphyte; Op, obligatory parasite; En/P, endophyte or facultative parasite; S, saprophyte.

Table S4 ITS-2 sequence percentages of fungi detected in air samples captured by a volumetric spore sampler at the experimental ash stand in 2010. Absence of data indicates that the given genus was not detected in the sample.

Systematic position	Genus	mode*	Date			
			**12.07.**	**19.07.**	**26.07.**	**09.08.**
p__Basidiomycota;Exobasidiomycetes;o__Entylomatales;f__Entylomataceae	*Tilletiopsis*	Ep	67.50	76.10	48.20	32.50
p__Ascomycota;Eurotiomycetes;o__Chaetothyriales;f__Herpotrichiellaceae	*Cladophialophora*	Ep	5.50	1.80	1.50	8.10
p__Basidiomycota;Tremellomycetes;o__Filobasidiales;f__Filobasidiaceae	*Cryptococcus*	Ep	1.90	0.50		1.40
p__Basidiomycota;Tremellomycetes;o__Tremellales;f__Incertae sedis	*Dioszegia*	Ep	0.30	0.60	0.90	1.40
p__Basidiomycota;Tremellomycetes;o__Tremellales;f__Incertae sedis	*Cryptococcus*	Ep	0.30		0.20	1.40
p__Basidiomycota;Microbotryomycetes;o__Sporidiobolales;f__Incertae sedis	*Sporobolomyces*	Ep	0.30	0.60	0.70	
p__Basidiomycota;Tremellomycetes;o__Cystofilobasidiales;f__Cystofilobasidiaceae	*Udeniomycetes*	Ep	0.60		0.20	
p__Basidiomycota;Tremellomycetes;o__Tremellales;f__Incertae sedis	*Bulleromycetes*	Ep			0.60	
p__Basidiomycota;Microbotryomycetes;o__Sporidiobolales;f__Sporidiobolaceae	*Sporidiobolus*	Ep		0.20	0.20	
p__Basidiomycota;Tremellomycetes;o__Cystofilobasidiales;f__Cystofilobasidiaceae	*Itersonilia*	Ep		0.10	0.20	
p__Basidiomycota;Tremellomycetes;o__Tremellales;f__Incertae sedis	*Bullera*	Ep		0.10	0.20	
p__Ascomycota;Eurotiomycetes;o__Chaetothyriales;f__Chaetothyriaceae	*Ceramothyrium*	Ep			0.20	
p__Basidiomycota;Heterobasidiomycetes;o__Christianseniales;f__None	*Cryptococcus*	Ep	0.30	0.10		
p__Ascomycota;Eurotiomycetes;o__Chaetothyriales;f__Herpotrichiellaceae	*Exophiala*	Ep		0.10		
**SUM**			76.70	80.20	53.10	44.80
Exobasidiomycetes;o__Exobasidiales;f__Exobasidiaceae;g__Exobasidium	*Exobasidium*	Op	0.60	0.30		1.40
Leotiomycetes;o__Erysiphales;f__Erysiphaceae;g__Phyllactinia	*Phyllactinia*	Op		0.20		1.40
**SUM**			0.60	0.50		2.80
p__Ascomycota;Dothideomycetes;o__Capnodiales;f__Davidiellaceae	*Cladosporium*	En/P	3.20	1.00	6.80	16.20
p__Ascomycota;Leotiomycetes;o__Helotiales;f__Helotiaceae	*Hymenoscyphus*	En/P	1.00	8.50	9.00	8.10
p__Ascomycota;Dothideomycetes;o__Pleosporales;f__Incertae sedis;	*Peyronellae*	En/P	0.30	0.10	0.40	1.40
p__Ascomycota;Dothideomycetes;o__Incertae sedis;f__Incertae sedis	*Leptospore*	En/P	1.00	0.20	0.70	
p__Ascomycota;Dothideomycetes;o__Pleosporales;f__Phaeosphaeriaceae	*Phaeospheria*	En/P		0.10	0.20	1.40
p__Ascomycota;Dothideomycetes;o__Capnodiales;f__Davidiellaceae	*Davidiella*	En/P				1.40
p__Ascomycota;Leotiomycetes;o__Helotiales;f__Helotiaceae	*Crocicreas*	En/P			0.90	
p__Ascomycota;Leotiomycetes;o__Helotiales;f__Helotiaceae	*Articulospora*	En/P	0.30		0.20	
p__Ascomycota;Dothideomycetes;o__Pleosporales;f__Leptosphaeriaceae	*Leptosphaeria*	En/P		0.10	0.20	
p__Ascomycota;Leotiomycetes;o__Helotiales;f__Incertae sedis	*Cadophora*	En/P	0.30			

p__Ascomycota;Sordariomycetes;o__Xylariales;f__Xylariaceae	*Rosellinia*	En/P	0.30			
p__Ascomycota;Dothideomycetes;o__Pleosporales;f__Incertae sedis	*Boeremia*	En/P			0.20	
p__Ascomycota;Dothideomycetes;o__Pleosporales;f__Incertae sedis	*Didymella*	En/P			0.20	
p__Ascomycota;Dothideomycetes;o__Pleosporales;f__Incertae sedis	*Phoma*	En/P		0.20		
p__Ascomycota;Dothideomycetes;o__Pleosporales;f__Pleosporaceae	*Pleospora*	En/P			0.20	
p__Ascomycota;Dothideomycetes;o__Pleosporales;f__Pleosporaceae	*Pyrephora*	En/P			0.20	
p__Ascomycota;Leotiomycetes;o__Helotiales;f__Incertae sedis	*Leptodontidium*	En/P			0.20	
p__Ascomycota;Leotiomycetes;o__Helotiales;f__Incertae sedis	*Naevala*	En/P			0.20	
p__Ascomycota;Leotiomycetes;o__Helotiales;f__Incertae sedis	*Rhexocercosporidium*	En/P			0.20	
p__Ascomycota;Dothideomycetes;o__Dothideales;f__Dothioraceae	*Aureobasidium*	En/P		0.10		
p__Ascomycota;Dothideomycetes;o__Pleosporales;f__Incertae sedis	*Setomelanomma*	En/P		0.10		
p__Ascomycota;Leotiomycetes;o__Helotiales;f__Incertae sedis	*Tetracladium*	En/P		0.10		
p__Ascomycota;Leotiomycetes;o__Helotiales;f__Sclerotiniaceae	*Botryotinia*	En/P		0.10		
p__Ascomycota;Sordariomycetes;o__Hypocreales;f__Nectriaceae	*Fusarium*	En/P		0.10		
**SUM**			6.40	10.70	19.80	28.50
p__Ascomycota;Eurotiomycetes;o__Eurotiales;f__Trichocomaceae	*Penicillium*	S	1.00	0.10		
p__Ascomycota;Leotiomycetes;o__Helotiales;f__Dermateaceae	*Mollisia*	S		0.10	0.40	
p__Ascomycota;Eurotiomycetes;o__Chaetothyriales;f__Herpotrichiellaceae	*Phialophora*	S			0.40	
p__Ascomycota;Eurotiomycetes;o__Chaetothyriales;f__Herpotrichiellaceae	*Rhinocladiella*	S		0.10		
p__Basidiomycota;Agaricomycetes;o__Polyporales;f__Meruliaceae	*Steccherinum*	S	1.30	0.40	0.60	4.10
p__Basidiomycota;Agaricomycetes;o__Russulales;f__Bondarzewiaceae	*Heterobasidion*	S	5.10		0.40	
p__Basidiomycota;Agaricomycetes;o__Russulales;f__Lachnocladiaceae	*Scytinostroma*	S	0.30		2.40	2.70
p__Basidiomycota;Agaricomycetes;o__Polyporales;f__Meruliaceae	*Phlebia*	S	0.00		0.20	1.40
p__Basidiomycota;Agaricomycetes;o__Polyporales;f__Meruliaceae	*Scopuloides*	S	0.00		0.20	1.40
p__Basidiomycota;Agaricomycetes;o__Hymenochaetales;f__Schizoporaceae	*Xylodon*	S	0.60	0.70	0.20	
p__Basidiomycota;Agaricomycetes;o__Polyporales;f__Meripilaceae	*Grifola*	S	1.30			
p__Basidiomycota;Agaricomycetes;o__Hymenochaetales;f__Schizoporaceae	*Other*	S	0.00		0.70	
p__Basidiomycota;Agaricomycetes;o__Hymenochaetales;f__Hymenochaetaceae	*Phellinus*	S	0.00	0.40	0.20	
p__Basidiomycota;Agaricomycetes;o__Russulales;f__Peniophoraceae	*Peniophora*	S	0.00	0.20	0.40	
p__Basidiomycota;Agaricomycetes;o__Polyporales;f__Polyporaceae	*Polyporus*	S	0.00	0.20	0.20	
p__Basidiomycota;Agaricomycetes;o__Polyporales;f__Polyporaceae	*Lentinus*	S	0.00		0.20	
p__Basidiomycota;Agaricomycetes;o__Agaricales;f__Mycenaceae	*Mycena*	S	0.00	0.20		
**SUM**			9.60	2.40	6.50	9.60

*Putative feeding mode: Ep, epiphyte; Op, obligatory parasite; En/P, endophyte or facultative parasite; S, saprophyte.

Table S5 Pearson product moment correlation between ITS-2 sequence proportions of *Hymenoscyphus fraxineus* and the most common fungal general in different ash leaf tissues across the season 2011. Statistically significant correlations are indicated by ** ($P < 0.01$) or by * ($P < 0.05$).

Putative mode	Genus	Leaflet	Petiole_upper	Petiole_base	All tissues^
Ep#	*Bullera*	0.48	0.32	0.31	0.38
Ep	*Cryptococcus*	0.19	0.35	-0.37	0.04
Ep	*Dioszegia*	0.27	0.30	-0.32	0.06
Ep	*Rhodotorula*	-0.49	0.27	-0.04	-0.20
Ep	*Sporobolomyces*	-0.42	-0.45	-0.75*	-0.51**
Ep	*Tilletiopsis*	-0.65	-0.73*	-0.92**	-0.61**
Ep	*Aureobasidium*	-0.77**	-0.39	-0.85**	-0.67**
Ep	*Cladophialophora*	-0.48	-0.19	-0.32	-0.28
Ep	*Ceramothyrium*	-0.36	-0.34	0.02	-0.05
Op	*Exobasidium*	0.20	0.73*	0.29	0.23
Op	*Phyllactinia*	0.88**	-0.65	0.06	0.41
Op	*Taphrina*	-0.42	-0.45	-0.75*	-0.51**
Op	*Entyloma*	-0.08	-0.34	-0.20	-0.19
En/P	*Acicuseptoria*	0.47	-0.03	0.03	0.16
En/P	*Cladosporium*	-0.11	0.30	0.02	0.07
En/P	*Devriesia*	0.51	0.43	0.61	0.41*
En/P	*Epicoccum*	0.25	0.02	-0.11	0.07
En/P	*Fusarium*	0.45	0.56	0.35	0.34
En/P	*Knufia*	0.81*	0.80*	0.56	0.72**
En/P	*Leptosphaeria*	-0.31	-0.01	-0.08	-0.17
En/P	*Naevala*	-0.24	-0.16	-0.84	-0.15
En/P	*Paraleptosphaeria*	-0.53	0.07	-0.17	-0.19
En/P	*Peyronellaea*	0.46	-0.03	-0.20	0.06
En/P	*Phoma*	0.34	0.39	0.51	0.43*
En/P	*Pleospora*	0.87	0.64	-0.96*	0.39

#

^All the three leaf tissues were pooled together.

#Putative feeding mode based on literature: Ep, epiphyte; Op, obligatory parasite; En/P endophyte or facultative parasite.

Fig. S6 Portion of nucleotide alignment of *Hymenoscyphus* ITS-2 sequences including a putative new undescribed genotype of *H. fraxineus* (Individual #1 in alignment). The 'CG' base pair differences indicated occurs at 85–86 base pairs downstream from the gITS-7 primer site in the 5.8S region. The other *Hymenoscyphus* individuals include four *H. fraxineus* and two *H. albidus* variants (Genbank references are A: JQ658347, B: JQ658348, C: JQ658349, D: JQ658350, E: JQ658351, F: JQ658352) sequenced from Norwegian samples in our laboratory and published in Hietala *et al*. (2013), as well as four sequences downloaded from Genbank (Genbank IDs GU586904, GU586876, AY789432, and JX977150).

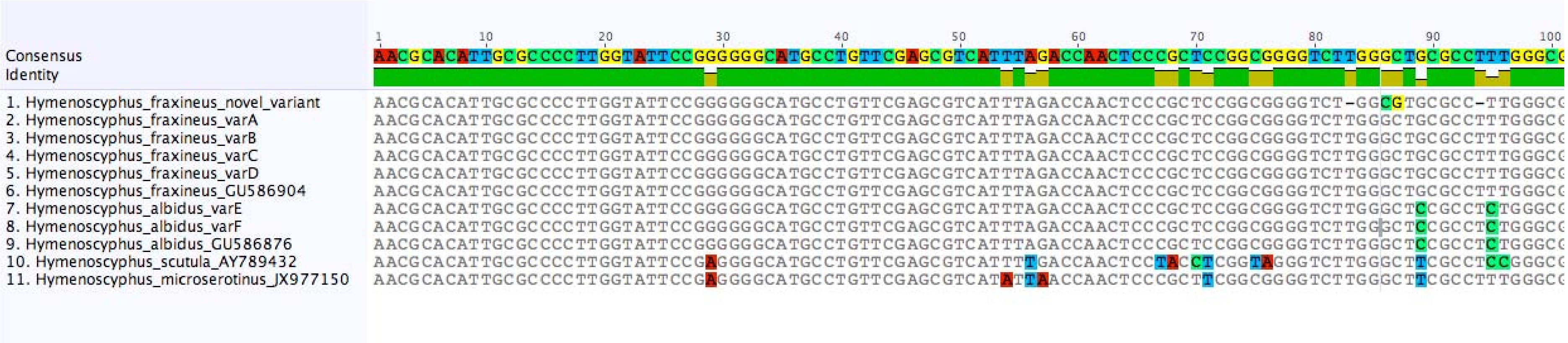

Fig. S7 Seasonal changes in *H. fraxineus* DNA amount, determined by qPCR and by ITS-2 read percentages of the two main sequence variants assigned to this species: variant1 shows 100% sequence identity to the European type specimen of *H. fraxineus* (FJ597975) and 98.8% sequence similarity to variant2. For qPCR the values represent means of tissue samples from three trees per each time point. Before sequencing the three replicates per tissue type and time point were pooled together.

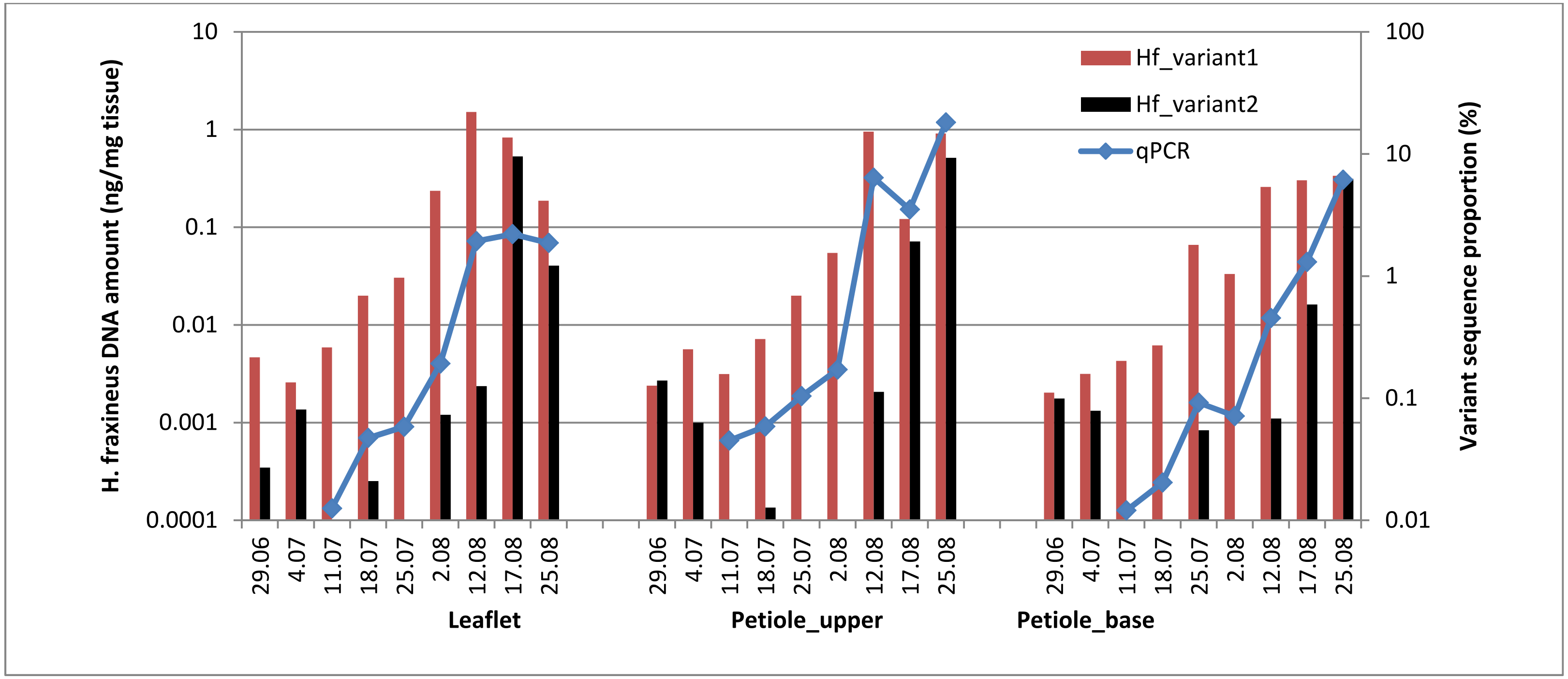

Supplement: Supplementary file 1 — Fig. S1 Monthly precipitation and mean temperature in 2011 and 2012. Fig. S2 Damage assessment of sampled ash trees in early (22 June) and late (23 August) summer 2012. Fig. S3 Hymenoscyphus fraxineus DNA amount in tissues collected from healthy and diseased ash trees throughout the summer 2012 and analyzed by a qPCR assay specific to the DNA of the fungus. Fig. S4 Alpha rarefaction curves of individual samples for next generation sequencing ITS‐1 and ITS‐2 datasets. Fig. S5 Seasonal changes in ITS‐1 and ITS‐2 read proportions of fungal taxa at the experimental stand. Fig. S6 Portion of nucleotide alignment of Hymenoscyphus ITS‐2 sequences including a putative new undescribed genotype of H. fraxineus. Fig. S7 Seasonal changes in H. fraxineus DNA amount, determined by qPCR and by ITS‐2 read percentages of the two main sequence variants assigned to this species. Table S1 The most common fungal genera present in both ITS‐1 and ‐2 datasets, and their sequence proportions (%) in leaf tissues in early summer (29 June–11 July), mid‐summer and late summer (12–25 August) in 2011 Table S2 The most common fungal genera detected in one ITS dataset only and their sequence proportions in leaf tissues in early summer (29 June–11 July), mid‐summer (18 July–02 August) and late summer (12–25 August) in 2011 Table S3 ITS‐1 sequence percentages of fungi detected in air samples captured by a volumetric spore sampler at the experimental ash stand in 2010 Table S4 ITS‐2 sequence percentages of fungi detected in air samples captured by a volumetric spore sampler at the experimental ash stand in 2010 Table S5 Pearson product moment correlation between ITS‐2 sequence proportions of Hymenoscyphus fraxineus and the most common fungal general in different ash leaf tissues across the season 2011 Methods S1 Detailed laboratory, bioinformatics and statistical analyses. [file NPH-213-1405-s001.pdf]
